# Multivesicular Body Formation Requires OSBP–Related Proteins and Cholesterol

**DOI:** 10.1371/journal.pgen.1001055

**Published:** 2010-08-05

**Authors:** Hiroyuki Kobuna, Takao Inoue, Machiko Shibata, Keiko Gengyo-Ando, Akitsugu Yamamoto, Shohei Mitani, Hiroyuki Arai

**Affiliations:** 1 Graduate School of Pharmaceutical Sciences, University of Tokyo, Tokyo, Japan; 2 Core Research for Evolutional Science and Technology (CREST), Japan Science and Technology Agency (JST), Tokyo, Japan; 3 Department of Physiology, Tokyo Women's Medical University School of Medicine, Tokyo, Japan; 4 Department of Bio-Science, Nagahama Institute of Bio-Science and Technology, Nagahama, Japan; University of California San Francisco, United States of America

## Abstract

In eukaryotes, different subcellular organelles have distinct cholesterol concentrations, which is thought to be critical for biological functions. Oxysterol-binding protein-related proteins (ORPs) have been assumed to mediate nonvesicular cholesterol trafficking in cells; however, their *in vivo* functions and therefore the biological significance of cholesterol in each organelle are not fully understood. Here, by generating deletion mutants of ORPs in *Caenorhabditis elegans*, we show that ORPs are required for the formation and function of multivesicular bodies (MVBs). In an RNAi enhancer screen using *obr* quadruple mutants (*obr-1; -2; -3; -4*), we found that MVB–related genes show strong genetic interactions with the *obr* genes. In *obr* quadruple mutants, late endosomes/lysosomes are enlarged and membrane protein degradation is retarded, although endocytosed soluble proteins are normally delivered to lysosomes and degraded. We also found that the cholesterol content of late endosomes/lysosomes is reduced in the mutants. In wild-type worms, cholesterol restriction induces the formation of enlarged late endosomes/lysosomes, as observed in *obr* quadruple mutants, and increases embryonic lethality upon knockdown of MVB–related genes. Finally, we show that knockdown of ORP1L, a mammalian ORP family member, induces the formation of enlarged MVBs in HeLa cells. Our *in vivo* findings suggest that the proper cholesterol level of late endosomes/lysosomes generated by ORPs is required for normal MVB formation and MVB–mediated membrane protein degradation.

## Author Summary

The multivesicular body (MVB) sorting pathway provides a mechanism for the lysosomal degradation of membrane proteins, such as growth factor receptors. The formation of MVBs is unique in that the curvature is directed toward the lumen of the compartment rather than the cytosol. During MVB formation, the curvature-inducing proteins, such as clathrins, could not be involved in the inward invagination of the endosomal membrane. Under these circumstances, lipids have been assumed to play a role in the membrane invagination step by creating local membrane environments; however, the lipids involved in this step have not been fully elucidated. Here we demonstrate that cholesterol, an essential membrane component in animals, is critical for MVB formation and function. We found that disruption of OSBP–related proteins (ORPs), which have been proposed to function in cellular cholesterol distribution and metabolism, reduces the cholesterol content in late endosomes/lysosomes, leading to impaired MVB function. MVB sorting pathway is known to be involved in many processes, including growth factor receptor down-regulation, exosome secretion, antigen presentation, the budding of enveloped viruses, and cytokinesis. Our findings provide a novel link between cholesterol and these biologically important functions.

## Introduction

The multivesicular body (MVB) sorting pathway provides a mechanism for the lysosomal degradation of membrane proteins and has a role in many processes, including growth factor receptor down-regulation [1], antigen presentation [2], developmental signaling [3], [4], the budding of enveloped viruses [5], and cytokinesis [6], [7]. MVBs form when the limiting membrane of the late endosomes invaginates and buds into the lumen of the organelle, selecting a subset of the proteins from the limiting membrane in the process [8], [9]. The MVB sorting machinery is constituted by proteins that form the endosomal sorting complexes required for transport (ESCRT-I, -II, and -III) [10], [11]. These ESCRT complexes are recruited sequentially to endosomal membranes where they function in sorting cargo and generating characteristic intralumenal vesicles. MVBs then fuse with lysosomes, resulting in degradation of their cargo. In addition to the ESCRT proteins, lipid molecules have been assumed to be involved in MVB formation by creating local microdomains in the endosomal membrane that induce the inward membrane curvature. For example, lysobisphosphatidic acid (LBPA) and ceramide were shown to induce the formation of internal vesicles in liposomes [12], [13]. Furthermore, treatment with anti-LBPA antibodies disrupts normal MVB formation in mammalian cells, suggesting that LBPA has a role in driving lumenal-vesicle formation at the cellular level [14].

In eukaryotes, different organelles within a cell generally have distinct cholesterol concentrations. Such differences are thought to be necessary for various biological functions ranging from membrane trafficking to signal transduction [15]. Obtaining the normal subcellular cholesterol distribution is thought to require a variety of intracellular cholesterol movements through vesicular and nonvesicular mechanisms [16], [17]. Recently, oxysterol-binding protein (OSBP) and OSBP-related proteins (ORPs) have been shown to mediate a number of cellular processes including signal transduction, lipid metabolism, vesicular trafficking and nonvesicular sterol transport [18]–[20]. OSBP was first identified as a high-affinity cytosolic receptor for oxysterols, such as 25-hydroxycholesterol [21]. Subsequently, most eukaryotes have been shown to have proteins homologous to OSBP, including 12 ORP-homologs in humans (OSBP and ORP1 to ORP11), four in *C. elegans* (this study; OBR-1 to OBR-4), four in *D. melanogaster*, and seven in the budding yeast *S. cerevisiae* (Osh1p to Osh7p) [19], [22]. Most ORPs share two highly homologous structural features: a PH domain at the amino-terminus and a ∼400-amino acid sterol-binding domain at the carboxy-terminus (Figure S1) [19]. The mammalian ORP family can be subdivided into six subfamilies (I–VI) based on gene organization and amino acid homology. Yeast ORPs share comparatively low sequence homologies with mammalian ORP proteins and are not classified into the ORP subfamilies, whereas *C. elegans* and *D. melanogaster* ORPs clearly fall into subfamilies I, II, IV and V based on the homology of the sterol-binding domains (Figures S1, S3, S4, S5, S6).

Many lines of evidence suggest that ORPs have a role in sterol distribution among intracellular organelles. Raychaudhuri showed that yeast ORPs (Osh4p, Osh5p, and Osh3p) have a role in transporting sterol from the yeast plasma membrane to the esterification compartment, ER [18]. In addition, the cholesterol distribution in yeast ORPs mutants was abnormal. A crystal structure analysis indicated that Osh4p is able to accommodate a variety of sterols including cholesterol [23]. In *in vitro* analyses, Osh4p and mammalian ORPs transferred sterols from donor to acceptor liposomes [18], [24]. In mammalian cells, the transport of newly synthesized cholesterol from the ER to the cell surface is enhanced by expression of ORP2 [25]. Although increasing evidence supports the involvement of ORP proteins in subcellular cholesterol distribution, knockout studies of ORPs in animals have not been reported, and consequently, the biological significance of distinct cholesterol concentrations in subcellular compartments remains to be elucidated.

In the present study, we generated deletion mutants of all ORP family members in *C. elegans* (*obr-1, -2, -3*, and *obr-4*). We also performed an RNAi modifier screen using *obr* quadruple mutants and found that a group of MVB-related genes including ESCRT complex genes show strong genetic interactions with *obr* genes.

## Results

### Generation of deletion mutants of *C. elegans* ORP family members

A database search revealed the presence of four ORP family members in *C. elegans*, which are classified into ORP subfamilies I, II, IV and V based on the homology of the sterol-binding domains. We named these ORP genes *obr-1*, *obr-2*, *obr-3*, and *obr-4*, respectively [*obr*: Oxysterol Binding protein (OSBP) Related (Figure S1, S3, S4, S5, S6) [19]]. To address the functions of ORP members, we generated deletion mutants of all four ORP genes in *C. elegans* by PCR-based screening of TMP/UV-mutagenized libraries (Figure S2) [26]. All of these mutations appear to be null or strong loss-of-function alleles because inhibition of each *obr* gene by RNAi failed to enhance the *obr* quadruple mutant phenotypes, such as embryonic lethality and slow growth as described below.

Single mutant worms with deletions in *obr-1*, *obr-2*, *obr-3*, or *obr-4* were viable and fertile, and displayed an essentially normal phenotype under a dissection microscope (Table 1). The *obr-1;obr-2;obr-3;obr-4* quadruple mutants that lacked all *obr* genes exhibited embryonic lethality (∼11%) and slow growth during larval development (∼18%) (Table 1). Hatched *obr* quadruple mutants were able to develop to adults and produce subsequent progeny, although they had a reduced brood size (60% of that of wild-type worms) and showed abnormal cuticle structure (Figure S7B and S7D). These data indicate that four *C. elegans* ORP proteins act redundantly during embryonic and larval development. This is similar to the case in yeast where any one of the 7 ORPs is sufficient for viability [27].

**Table 1:** Phenotypic consequences of *obr* mutants.

Cholesterol in NGM	Genotype	Embryonic lethality (%)	Growth defect (%)
+	Wild-type	<3	<3
+	*obr-1(xh16)*	<3	<3
+	*obr-2(xh17)*	<3	<3
+	*obr-3(tm1087)*	<3	<3
+	*obr-4(tm1567)*	<3	<3
+	*obr-1(xh16); obr-2(xh17)*	<3	<3
+	*obr-1(xh16); obr-3(tm1087)*	<3	<3
+	*obr-1(xh16); obr-4(tm1567)*	<3	<3
+	*obr-2(xh17); obr-3(tm1087)*	<3	<3
+	*obr-2(xh17); obr-4(tm1567)*	<3	<3
+	*obr-3(tm1087); obr-4(tm1567)*	<3	<3
+	*obr-2(xh17); obr-3(tm1087); obr-4(tm1567)*	<3	<3
+	*obr-1(xh16); obr-3(tm1087); obr-4(tm1567)*	<3	11
+	*obr-1(xh16); obr-2(xh17); obr-4(tm1567)*	<3	<3
+	*obr-1(xh16); obr-2(xh17); obr-3(tm1087)*	<3	<3
+	*obr-1(xh16); obr-2(xh17); obr-3(tm1087); obr-4(tm1567)*	11	18
−	Wild-type	<3	<3
−	*obr-1(xh16); obr-2(xh17); obr-3(tm1087); obr-4(tm1567)*	18	60

Data represent the mean among progeny of 10 individual animals.

### Knockdown of multivesicular body (MVB)–related genes enhances embryonic lethality of *obr* quadruple mutants

**Figure 1:**
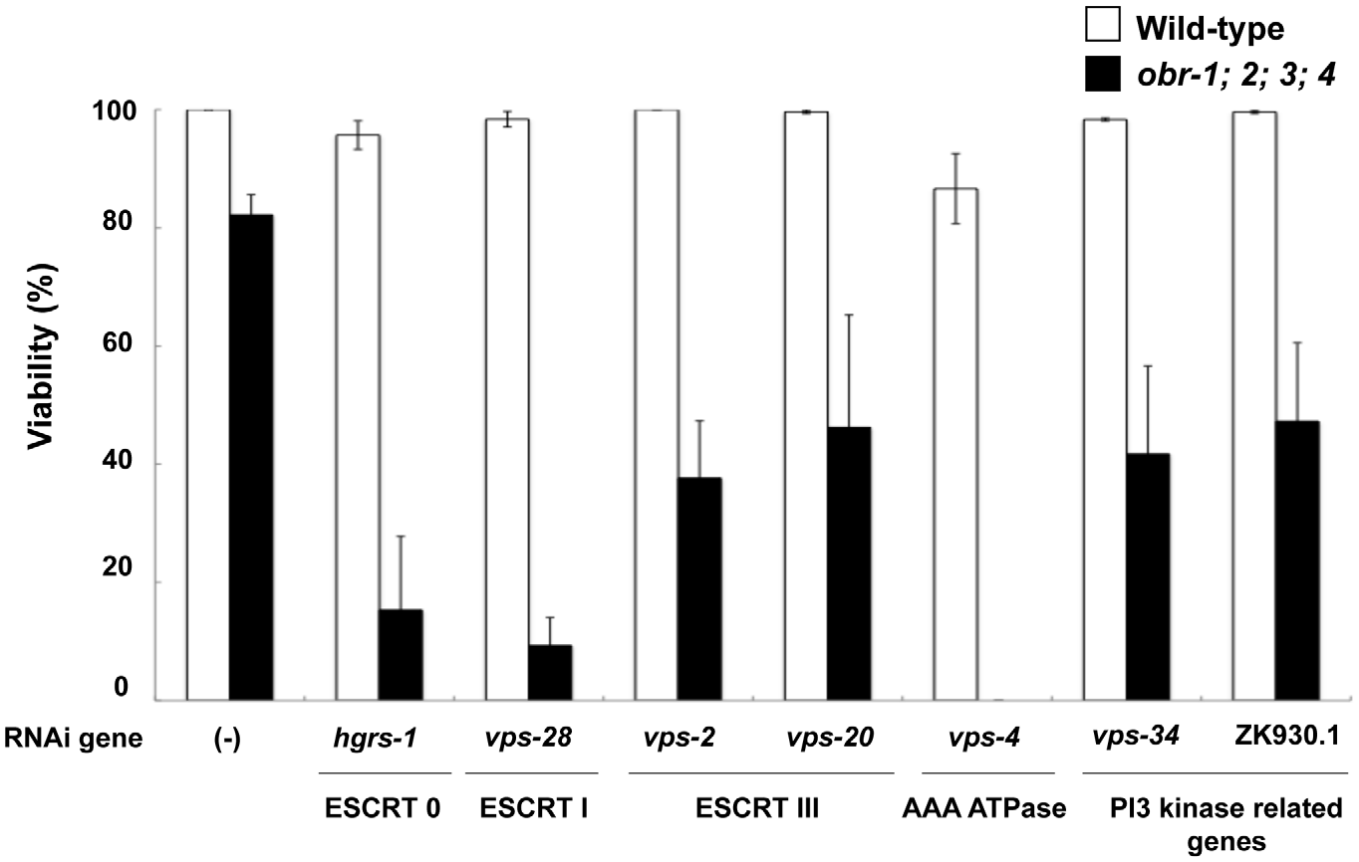
Synergism between *obr* genes and genes related to MVB formation in embryogenesis. The graph depicts the effects of suppression of MVB-related genes on the viability of wild-type and *obr* quadruple mutants (*obr-1;2;3;4*). Non-viable animals died at the embryo stage and were scored as described in Materials and Methods after feeding RNAi against the indicated genes. Error bars represent the standard error of the proportion.

To gain insights into the molecular mechanisms of embryonic lethality in *obr* quadruple mutants, we conducted a synthetic lethal screen. We used feeding RNAi clones on chromosomes I and III in the Ahringer library to identify RNAi clones that cause embryonic lethality in the *obr* quadruple mutant background, but not in the wild-type background (see Materials and Methods, and Table S1). As a result, we identified 28 genes that showed synthetic lethality in *obr* quadruple mutants (Table S2, hereafter, we refer to *obr-1;obr-2;obr-3;obr-4* quadruple mutants as the “*obr*s mutants”). These enhancer genes included genes encoding vesicular transport-related proteins, signaling proteins, and nuclear proteins. Interestingly, among the 28 enhancer genes, 6 genes (*hgrs-1*, *vps-28*, *vps-2*, *vps-20*, *vps-4*, and *vps-34*) have been reported to function in the formation of multivesicular bodies (MVBs), the machinery for degrading membrane proteins (Figure 1). Knockdown of *vps-4* caused complete embryonic lethality in the *obr*s mutants as compared to 13% embryonic lethality in wild-type animals (Figure 1). RNAi against other enhancer genes (*hgrs-1*, *vps-28*, *vps-2*, *vps-20*, and *vps-34*) also showed remarkably increased embryonic lethality (50–80%) in the *obr*s mutants as compared to wild-type worms (0–10%) under the present feeding RNAi conditions (Figure 1). In the *eri-1(mg366); lin-15B(n744)* background, which is hypersensitive to RNAi, knockdown of *vps-4*, *hgrs-1*, *vps-28* or *vps-32.2* (a component of ESCRT-III) resulted in embryonic lethality with high penetrance in wild-type worms, indicating that ESCRT components are essential for embryonic development in *C. elegans* [28] (data not shown).

Formation of MVBs requires the components of four complexes that include Vps27 (sometimes referred to as ESCRT-0), ESCRT-I, ESCRT-II, and ESCRT-III [10]. These complexes are recruited sequentially to endosomal membranes where they function in sorting cargo and generating intralumenal vesicles. The 6 *obr* enhancer genes encode *C. elegans* homologues of the ESCRT components or their regulatory molecules. These include *hgrs-1*, a homologue of yeast Vps27, *vps-28*, a component of ESCRT-I, *vps-2* and *vps-20*, components of ESCRT-III, *vps-4*, a homologue of yeast Vps4/AAA ATPase that is recruited by ESCRT-III to disassemble and recycle the ESCRT machinery, and *vps-34*, a class III phosphoinositide 3 (PI3) kinase required for recruitment of ESCRT-0 to early endosomal membranes. ZK930.1, a homologue of mammalian p150 that encodes the PI3 kinase regulatory subunit, was also identified as an *obr* enhancer gene (Figure 1; Table S2).

### *obr* quadruple mutants exhibit enlarged late endocytic compartments

**Figure 2:**
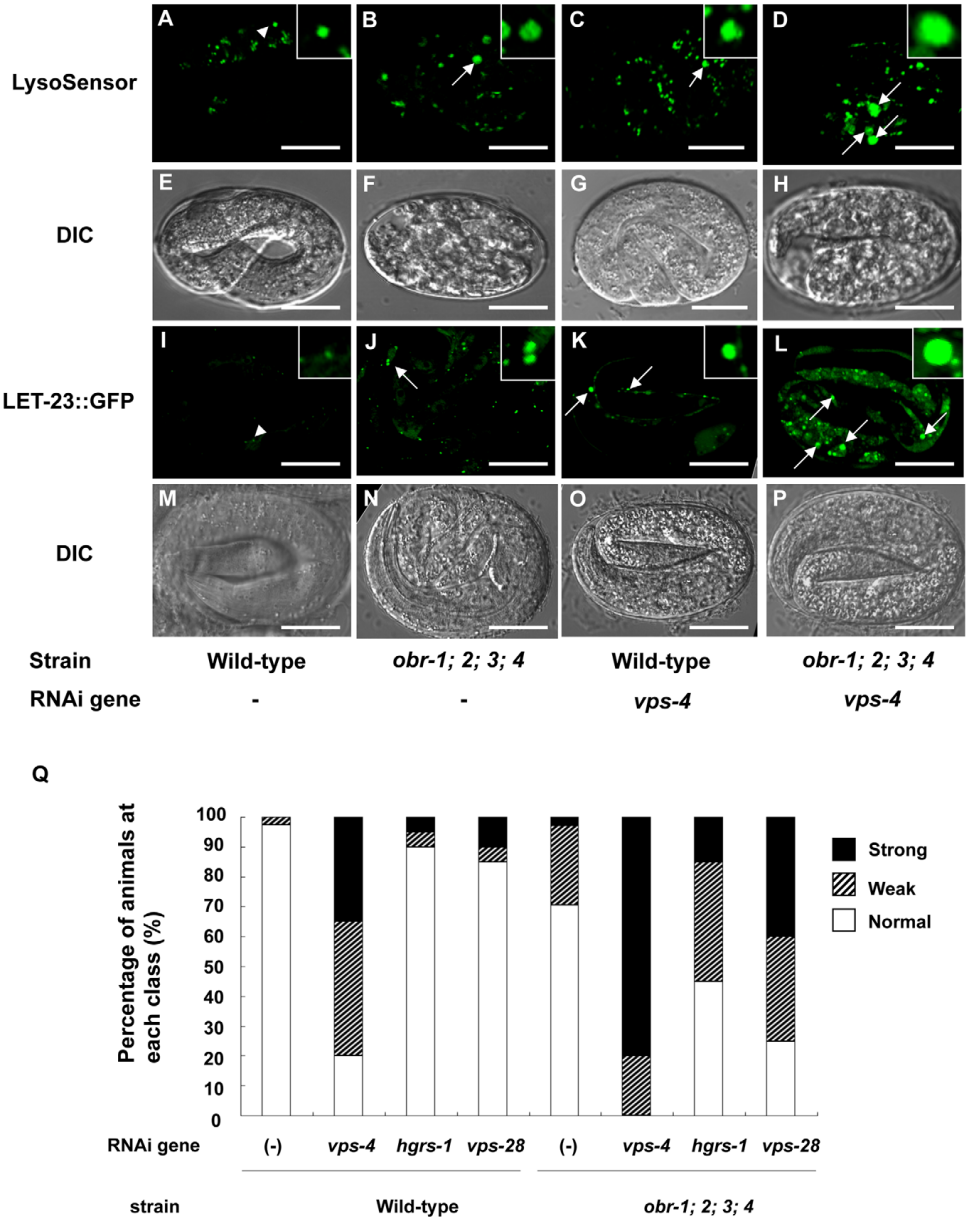
*obr* quadruple mutants contain enlarged lysosomes and accumulate LET-23::GFP, a *C. elegans* EGF receptor. (A–H) Enlarged lysosomes accumulate in *obr* quadruple mutants. Confocal fluorescence images and the corresponding Nomarski micrographs of 3-fold embryos laid by wild-type (A and E), *obr* quadruple mutants (*obr-1;2;3;4*) (B and F), wild-type subjected to *vps-4* RNAi (C and G), and *obr* quadruple mutants subjected to *vps-4* RNAi (D and H). Strains were grown on plates containing Lysosensor Green. Arrowhead indicates the Lysosensor Green-labeled lysosomes (A), and arrows indicate enlarged lysosomes (B, C and D). Insets are magnified three times. (I–P) LET-23::GFP accumulates in *obr* quadruple mutant embryos. Confocal fluorescence images and the corresponding Nomarski micrographs of embryos laid by LET-23::GFP-expressing wild-type (I and M), *obr* quadruple mutants (J and N), wild-type subjected to *vps-4* RNAi (K and O), and *obr* quadruple mutants subjected to *vps-4* RNAi (L and P). Arrowhead indicates the LET-23::GFP-positive endosomes/lysosomes (I), and arrows indicate the enlarged LET-23::GFP-positive vesicles (J, K and L). All images of the same marker were taken with the same exposure and at the same magnification (A–P: scale bars are 10 µm). Insets are magnified five times. (Q) Synergism between *obr* genes and MVB-related genes (*vps-4*, *hgrs-1*, *vps-28*). Diameter of LET-23::GFP-positive vesicles is classified into three categories: Normal (normal: <0.7 µm), Weak (weakly enlarged vesicle: 0.7–1.5 µm) and Strong (strongly enlarged: >1.5 µm). Graph shows the percentage of worms containing each category of LET-23::GFP-positive vesicles.

A strong genetic interaction between *obr* genes and MVB-related genes led us to hypothesize that late endocytic compartments (late endosomes/lysosomes) are affected in *obr*s mutants. In *S. cerevisiae*, disruption of an MVB-related gene, such as *vps-4* or *vps-28*, causes enlargement of aberrant late endocytic compartments and disturbance of membrane protein degradation [29]. To assess the morphology of late endocytic compartments in *obrs* mutant embryos, we first used the fluorescent probe LysoSensor Green, which accumulates in acidic compartments because of protonation [30]. In wild-type embryos, the probe localized to small punctate vesicles throughout embryogenesis (Figure 2A). In contrast, in *obrs* mutant embryos, the number of large fluorescent vesicles increased, indicating that late endocytic compartments were enlarged in *obr*s mutant embryos (Figure 2B). Knockdown of *vps-4* in wild-type embryos also caused the appearance of similar large fluorescent vesicles as observed in *obr*s mutant embryos (Figure 2C), and these enlarged vesicles were synergistically increased in the *obr*s mutant background (Figure 2D, Figure S12A). These data indicate that in *obr*s mutant embryos, late endocytic compartments were enlarged and these morphological defects were enhanced by knockdown of the MVB-related genes.

We next examined the expression of LET-23, a *C. elegans* homologue of the epidermal growth factor (EGF) receptor which is known to be degraded via the MVB pathway [1]. In wild-type embryos, LET-23::GFP was observed mostly in small punctate vesicles (<0.7 µm) during embryonic morphogenesis (Figure 2I and 2Q). In *obr*s mutants, LET-23::GFP vesicles were enlarged and GFP intensity was stronger than that in wild-type embryos (Figure 2J). A portion of LET-23::GFP-positive vesicles colocalized with the LysoTracker-labeled endosomes/lysosomes (Figure S12G, S12H, S12I). Knockdown of *vps-4* in wild-type embryos also caused the appearance of large vesicles similar to those observed in *obr*s mutant embryos (Figure 2K and 2Q). These observations indicate that LET-23::GFP was partly localized to late endocytic compartments in *obr*s mutants, although it is possible that some of the LET-23::GFP was localized to compartments other than endosomes/lysosomes. In the *obr*s mutant background, the GFP level was synergistically increased by knockdown of an MVB-related gene such as *vps-4*, *hgrs-1* or *vps-28* (Figure 2L and 2Q). These results suggest that degradation of the EGF receptor LET-23 was retarded in *obr*s mutants and this defect was synergistically enhanced by knockdown of MVB-related genes.

### Lysosomal degradation of soluble proteins and the endocytosis pathway are not affected in *obr* quadruple mutants

**Figure 3:**
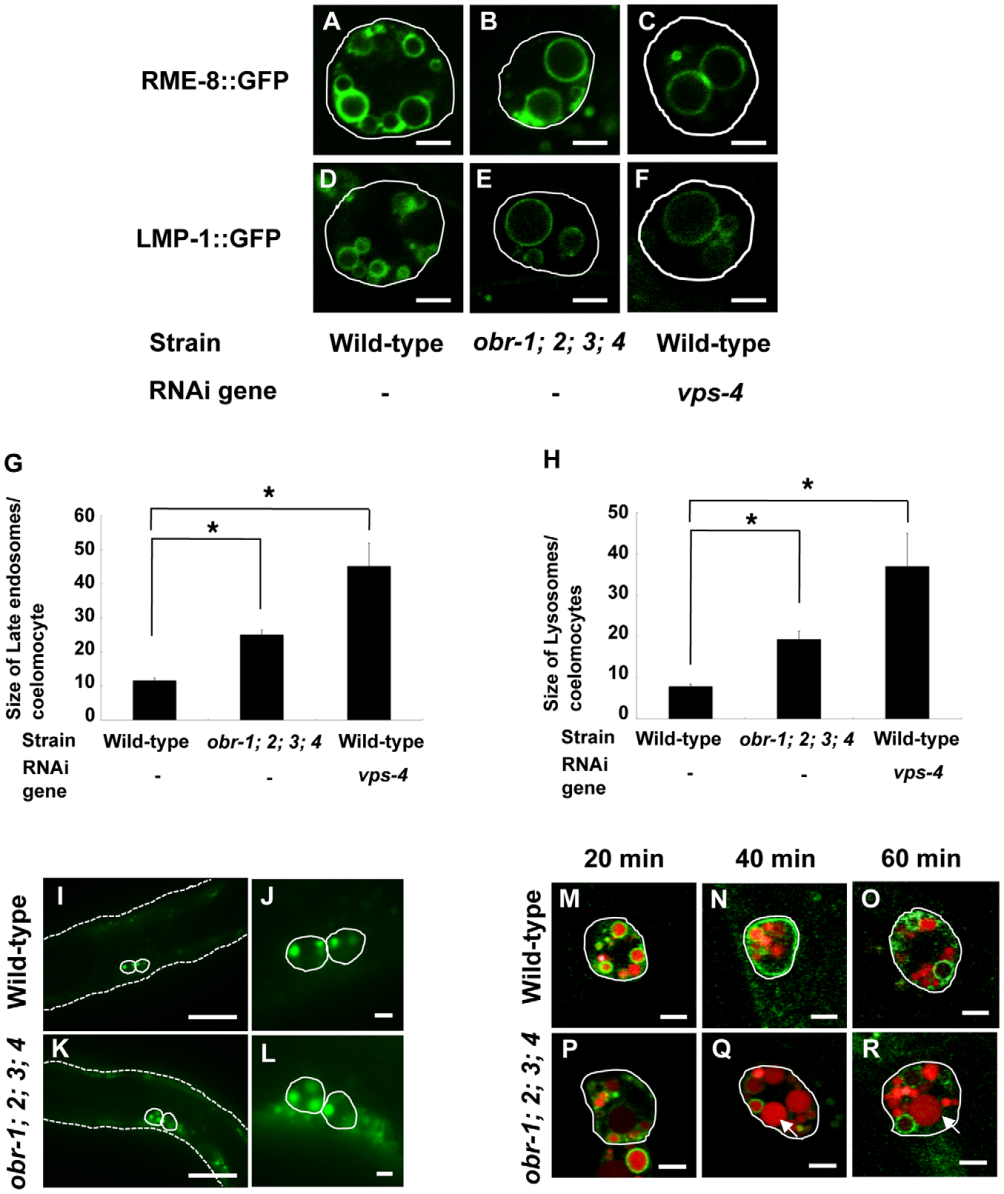
Morphological characterization of enlarged vacuoles in coelomocytes of *obr* quadruple mutants. (A–F) Confocal fluorescence images of different endocytic compartment markers in wild-type (A and D), *obr* quadruple mutants (*obr-1;2;3;4*) (B and E), and *vps-4 (RNAi)* coelomocytes (C and F). The outline of the coelomocyte is indicated by a white line. RME-8::GFP marks late endosomes (A–C), and LMP-1::GFP staining represent mostly lysosomes (D–F). Scale bars are 5 µm (A–F). (G and H) Quantification of the size of late endosomes and lysosomes in wild-type, *obr* quadruple mutants and *vps-4 (RNAi)* coelomocytes. (I–L) Confocal fluorescence images of wild-type (I and J) and *obr* quadruple mutants (K and L) expressing *myo-3p*::ssGFP. The left column shows worms at a low magnification (I and K), and the right column shows individual coelomocytes at a higher magnification (J and L). The outline of the worm and the coelomocyte is indicated by a dotted line (I and K) and by a white line (I–L), respectively. In *myo-3p*::ssGFP carrying worms, GFP is secreted from the muscle into the body cavity and subsequently, is endocytosed by the coelomocytes. Scale bars are 50 µm (I and K) or 5 µm (J and L). (M–R) Time course internalization of injected BSA-TR (Texas Red-conjugated BSA) through fluid-phase endocytosis in RME-8::GFP-expressing coelomocytes. The outline of the coelomocyte is indicated by a white line (M–R). (M and P) 20 minutes after injection of BSA-TR, BSA-TR appears within RME-8::GFP-positive late endosomes in wild-type and *obr* quadruple mutants. (N and Q) After 40 min, some TR-BSA fills RME-8::GFP-labeled vesicles at a low level, whereas some TR-BSA is concentrated into RME-8::GFP-negative vesicles in wild-type and *obr* quadruple mutants. (O and R) After 60 min, most of the BSA-TR appears in RME-8::GFP-negative lysosomes in wild-type and *obr* quadruple mutants. Note that RME-8::GFP-negative lysosomes are enlarged in *obr* quadruple mutant coelomocytes (Q and R, arrows). Scale bars are 5 µm (M–R).

We then investigated intracellular transport of soluble proteins endocytosed from the extracellular fluid (the body cavity) to late endocytic compartments in coelomocytes, scavenger cells that are highly active in endocytosis [31]. We first examined the morphology of endosomes and lysosomes in coelomocytes, and found that RME-8-labeled late endosomes (Figure 3A–3C) [32] and LMP-1-labeled lysosomes (Figure 3D–3F) [33] were significantly enlarged in *obr*s mutants. Enlargement of late endosomes/lysosomes was also observed in *vps-4*, *vps-2* or *hgrs-1* RNAi worms (Figure 3C, 3F–3H, and data not shown). These data are in agreement with the previous results showing enlargement of LysoSensor Green-positive vesicles in *obr*s mutant embryos (Figure 2B–2D and Figure S12A). In contrast, there appeared to be no differences in fluorescence patterns of early endosomes (2x FYVE::GFP), Golgi (AMAN-2::GFP), endoplasmic reticulum (GFP::TRAM) between wild-type and *obr*s mutants (Figure S9A, S9B, S9C, S9D, S9E, S9F, S9G).

Next, we investigated the fluid-phase endocytosis in *obr* mutants using a transgenic strain that secretes GFP from the muscle into the body cavity (*myo-3p::ss*GFP) [31]. In wild-type animals, the secreted soluble GFP (ssGFP) was rapidly endocytosed by the coelomocytes and degraded (Figure 3I and 3J) [31]. However, mutants defective in endocytosis or intracellular transport of endocytosed soluble proteins in coelomocytes showed increased levels of ssGFP in the body cavity [31]. In the *obr*s mutants, ssGFP appeared to be efficiently endocytosed by coelomocytes, producing animals with bright green coelomocytes as observed in wild-type worms (Figure 3K and 3L). To obtain higher temporal resolution, we microinjected Texas-red BSA into the body cavity of the worms [31], [34]. In wild-type worms, 20 min after injection, the marker started accumulating in the late endosomes of coelomocytes as indicated by the RME-8::GFP-positive compartments (Figure 3M). By 60 min, it was observed increasingly in lysosomes but was absent from RME-8::GFP-positive late endosomes (Figure 3N and 3O). In *obr*s mutants, the fluid-phase endocytosis and postendocytic trafficking proceeded with the same kinetics as observed in wild-type worms (Figure 3P–3R). We also checked receptor-mediated endocytosis of a yolk protein VIT-2 in oocytes (Rme) [35], and found that VIT-2 was efficiently incorporated into oocytes in the *obr*s mutants in a similar manner to that in wild-type worms (Figure S8A and S8C). Taken together, these data indicate that endocytic trafficking of soluble proteins to lysosomes is not affected in *obr*s mutant coelomocytes.

### *obr* quadruple mutants are delayed in the degradation of membrane protein

**Figure 4:**
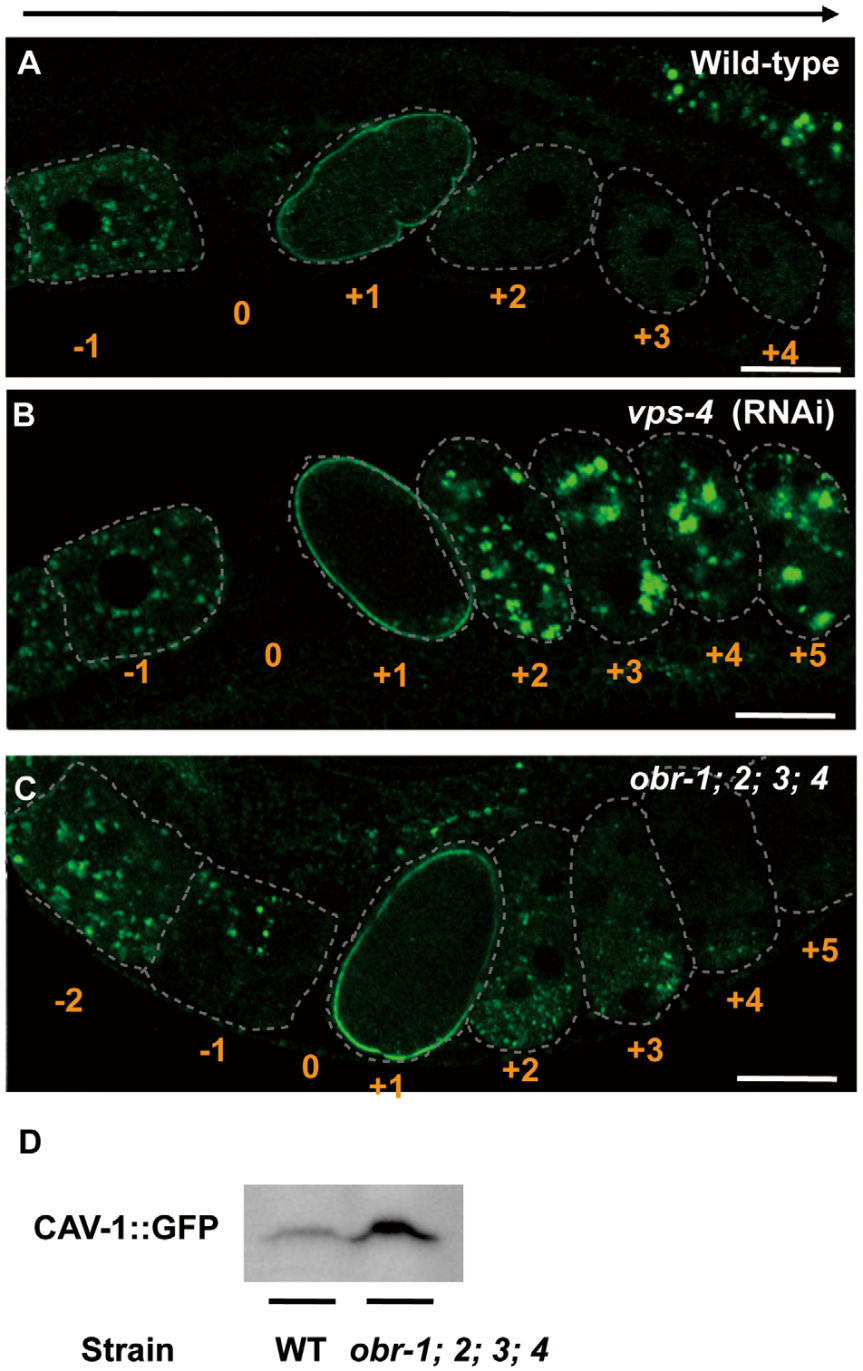
Depletion of *obr* genes slows the degradation of internalized CAV-1::GFP, but to a lesser extent than inhibition of the ESCRT components. (A–C) Pre-fertilization oocytes are numbered −1 and −2 with respect to their position relative to the spermatheca indicated by “0”. Similarly, fertilized embryos, which are at progressively later stages in development as their distance from the spermatheca increases, are numbered +1, +2, +3, etc. Confocal fluorescence images of wild-type (A), *vps-4 (RNAi)* worms (B), and *obr* quadruple mutants (C) expressing CAV-1::GFP. CAV-1 body formation appeared normal (“−1” embryos), but degradation of CAV-1::GFP was blocked or delayed in *vps-4 (RNAi)* worms and *obr* quadruple mutants, respectively. Arrows indicate the direction of maturation of oocytes and fertilized embryos. All scale bars are 20 µm. (D) Western blot detection of CAV-1::GFP in wild-type and *obr* quadruple mutants.

We next examined internalization and subsequent degradation of cell surface membrane proteins in the *obr*s mutants. To this end, we used a transgenic worm expressing a member of the caveolin protein family, CAV-1, that has been reported to be degraded via the MVB pathway during the oocyte-to-embryo transition [36]. In control oocytes prior to fertilization, CAV-1::GFP was concentrated in intracellular vesicles (Figure 4A, an oocyte indicated by “−1”) [37]. Immediately after oocytes passed through the spermatheca and were fertilized, the CAV-1::GFP signal of intracellular vesicles was lost and the CAV-1::GFP signal on plasma membrane rapidly increased (Figure 4A, an embryo indicated by “+1”). Most of CAV-1::GFP was internalized and degraded in the one-cell stage embryo and was not observed beyond the two-cell stage (Figure 4A, Figure S10A and S10B, embryos indicated by “+2” to “+4”). The post-fertilization increase in the amount of CAV-1::GFP on the cell surface and its subsequent re-internalization were not affected either in the *obr*s mutants or *vps-4* RNAi worms (“+1” and “+2” embryos in Figure 4A–4C). Consistent with previous results [36], knockdown of an MVB-related gene, such as *vps-4*, *hgrs-1*, *vps-28*, or *vps*-20, resulted in a substantial delay in the degradation of internalized CAV-1::GFP, which remained on internal membranes even in the “+5” embryo (an embryo at about the 26-cell stage) (Figure 4B and data not shown). The *obr*s mutants exhibited slightly but significantly retarded degradation of internalized CAV-1::GFP, where significant CAV-1::GFP signal was observed in intracellular membranes of +2 and +3 embryos (Figure 4C). A western blot analysis also revealed that the amount of CAV-1::GFP increased in the *obr*s mutants (Figure 4D). The milder defects in CAV-1::GFP degradation in the *obr*s mutants than in *vps-4* RNAi worms indicate that *obr* genes are not essential for the degradation of membrane proteins, but are required for efficient degradation of those proteins in *C. elegans* embryos.

### Knockdown of MVB–related genes causes high embryonic lethality under cholesterol-depleted conditions

**Figure 5:**
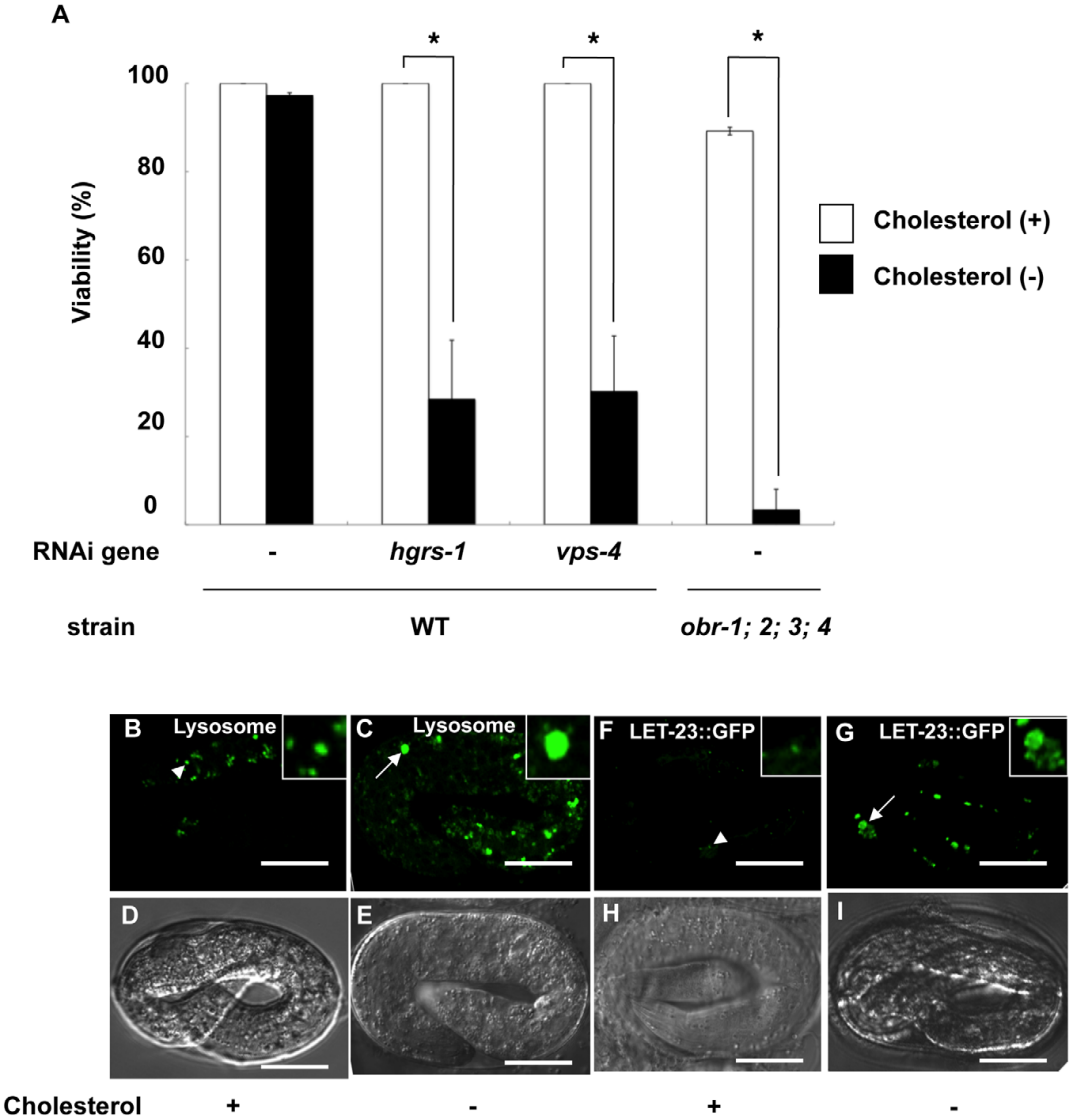
*obr* quadruple mutants and worms depleted of MVB formation-related genes by RNAi are hypersensitive to cholesterol depletion. (A) Viability of the progeny (F2) of the indicated strains which were kept under cholesterol-supplemented conditions (cholesterol+; open bar, Brenner condition) or under cholesterol-restricted conditions (cholesterol−; closed bar) (for details, see Materials and Methods). Non-viable animals died at the embryo stage. Error bars represent the standard error of the proportion. (B–E) Lysosomes are enlarged in wild-type embryos under cholesterol-restricted conditions. Confocal fluorescence images and the corresponding Nomarski micrographs of 3-fold embryos laid by wild-type grown on plates containing Lysosensor Green under cholesterol-supplemented conditions (B and D) and under cholesterol-restricted conditions (C and E). Arrowheads indicate Lysosensor-labeled lysosomes, and arrows indicate enlarged lysosomes. (F–I) LET-23::GFP accumulates in wild-type embryos under cholesterol-restricted conditions. Confocal fluorescence images and the corresponding Nomarski micrographs of embryos laid by LET-23::GFP-expressing wild-type worms under cholesterol-supplemented conditions (F and H) and under cholesterol-restricted conditions (G and I). Arrowheads indicate LET-23::GFP-positive vesicles (F), and arrows indicate enlarged LET-23::GFP vesicles (G). All scale bars are 10 µm. Insets are magnified three times.

Because ORPs have been implicated in intracellular cholesterol transport, we tested the possible involvement of cholesterol in MVB formation. *C. elegans* requires cholesterol for normal development, but does not possess the enzymes necessary for *de novo* sterol biosynthesis. Therefore *C. elegans* membrane cholesterol must be supplied by the diet [38]. The first generation of wild-type worms placed on cholesterol-depleted plates develop from eggs to adults without external cholesterol because cholesterol is supplied from mother worms grown on normal plates (Brenner condition; 5 µg/ml of cholesterol). However, 5% of second-generation embryos died (Figure 5A) and the development of all hatched larvae was arrested at the early larval stage (data not shown) [39]. Under these cholesterol-restricted conditions, second-generation *obr*s mutants exhibited 96% embryonic lethality whereas the mutants showed only 11% embryonic lethality under cholesterol-supplemented conditions (Figure 5A). The hypersensitivity of *obr*s mutants to cholesterol deprivation suggests that the OBR proteins are involved in the utilization of cholesterol in *C. elegans*.

We next performed knockdown of MVB-related genes under cholesterol-restricted conditions (see Materials and Methods). Under cholesterol-restricted conditions, knockdown of MVB-related genes, such as *hgrs-1* and *vps-4*, resulted in remarkably reduced viability and high penetrance embryonic lethality (Figure 5A). The reduced viability of *hgrs-1(RNAi)* and *vps-4(RNAi)* worms under cholesterol-restricted conditions is similar to that observed in the *obr*s mutant background (Figure 1). These results suggest that cholesterol content is critical for MVB formation during embryogenesis and that *obr* molecules regulate cholesterol content in *C. elegans*.

To examine whether the late endosomal/lysosomal defects observed in *obr*s mutants occur in wild-type worms under cholesterol-restricted conditions, we again used LysoSensor Green to visualize late endocytic compartments. As observed in *obr*s mutants (Figure 2B), late endocytic compartments were enlarged under the cholesterol-restricted conditions (Figure 5C). We also found that LET-23::GFP vesicles were enlarged and their GFP intensity was stronger under cholesterol-restricted conditions than under cholesterol-supplemented conditions (Figure 5F and 5G). These data indicate that cholesterol is essential for the normal morphology of late endocytic compartments and for the degradation of membrane proteins via MVB formation.

To examine the cholesterol content of the late endocytic compartments, wild-type and *obr*s mutants were fed with radioactive cholesterol and homogenized with a Dounce homogenizer device [40]. The crude membrane fraction (20,000×*g* ppt in Figure S11A) was subjected to density gradient centrifugation by using a Lysosome Isolation Kit (see Materials and Methods). ER and Golgi membranes were found in the high-density fractions (Figure S11A; fractions #1–4, PAF-2 and COGC-3, respectively) and late endosomes/lysosomes were recovered in the low-density fractions (Figure S11A; fractions #7, 8, RAB-7::GFP). In wild-type animals, appreciable amount of radioactive cholesterol was recovered in the late endosomal/lysosomal fractions (fractions #7 and #8), whereas the cholesterol content in the late endosomal/lysosomal fractions of the *obr*s mutants was approximately 75% of that of wild-type worms (Figure S11A and S11B). The total cholesterol content in *obr*s mutants was also reduced significantly (to ∼60% of that of wild-type, Figure S11C), indicating that ORPs are also important for determining the cholesterol content of *C. elegans*.

### ORP1L, a mammalian homologue of *obr-2*, is required for the formation of MVBs in HeLa cells

**Figure 6:**
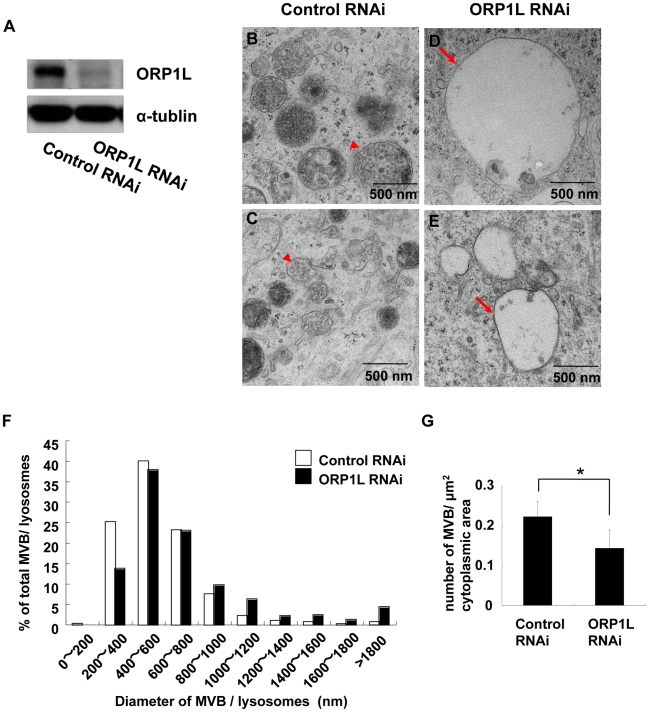
ORP1L depletion causes the enlargement of endosomes. (A) Western blots showing expression of ORP1L in ORP1L siRNA-treated HeLa cells. α-Tubulin was used as a protein loading control. (B–E) Control- and ORP1L siRNA-treated HeLa cells were analyzed by electron microscopy. In control cells, normal MVBs contain internal vesicles (B and C, arrowheads). In ORP1L-depleted cells, MVBs tend to be enlarged and contain fewer internal vesicles (D and E, arrows). (F) Histogram of diameters of late endosomes (n = 914) from control cells and late endosomes (n = 573) from ORP1L siRNA-treated cells. (G) Number of MVBs (mean ± SEM, n = 3) per 1 µm^2^ cytoplasmic area on the cell profiles of mock-treated and ORP1L RNAi-treated cells. Asterisk indicates a statistically significant difference (P<0.05).

Finally, we examined whether the functions of *C. elegans obr* members are conserved across species. We expressed all human ORP family members in HeLa cells and found that only ORP1L localized at lysosomes (data not shown) as reported previously [41]. ORP1L is structurally classified to ORP subfamily II which includes *C. elegans obr-2* (Figure S1 and Figure S4). To determine the effects of ORP1L depletion on late endosomal/lysosomal morphology, we analyzed the morphology at the ultrastructural level by electron microscopy. In control cells, late endosomal/lysosomal compartments appeared as relatively dense round structures of 0.2- to 1-µm diameter, in which numerous small vesicles (MVBs) could be seen (Figure 6B and 6C). In contrast, large swollen vacuoles of 0.6- to 1.8- µm diameter appeared in ORP1L siRNA-treated cells (Figure 6A and 6D–6F). These enlarged structures appeared to be MVBs because they still contained some intralumenal vesicles, although significantly less in number compared with the intralumenal vesicles in MVBs of control cells. Furthermore, ORP1L siRNA-treated cells had ∼30% less MVBs than control cells (Figure 6G). We next investigated whether depletion of ORP1L affects EGF receptor degradation (see Text S1). In HeLa cells treated with the control siRNA, the EGF receptor was gradually degraded after 1, 2, and 3 hr of EGF stimulation. siRNA against ORP1L delayed EGF-induced receptor degradation more than the control siRNA (Figure S13A and S13B). In conclusion, these results indicate that ORP1L is required for MVB formation, normal morphology of late endosomes/lysosomes and membrane protein degradation, and these functions are evolutionarily conserved in mammals.

## Discussion

### ORP proteins facilitate the formation of MVBs and subsequent membrane protein degradation

Cholesterol is a structural component of animal membranes that influences fluidity, permeability and formation of lipid microdomains. ORP family members have been implicated in the cholesterol distribution among intracellular organelles [18]–[25]; although their *in vivo* functions are not fully understood. In the present study, we generated deletion mutants of all ORP family members in *C. elegans* (*obr-1*, *-2*, *-3*, and *obr-4*) (Figure S2; Table 1). We also performed an RNAi modifier screen using *obr* quadruple mutants (*obr*s mutants) and found that a group of MVB-related genes including ESCRT complex genes show strong genetic interactions with *obr* genes (Figure 1; Table S2).

In *obr*s mutants, degradation of membrane proteins, such as an EGF receptor (LET-23::GFP) (Figure 2I–2L) and caveolin (CAV-1::GFP) (Figure 4), is delayed and late-endosomes/lysosomes are enlarged (embryos; Figure 2B, coelomocytes; Figure 3B and 3E). At the ultrastructural level, *obr*s mutants have enlarged vacuoles which are not observed in wild-type worms (Figure S7A and S7B). Similar defects of endocytic compartment have been reported in ESCRT-depleted *S. cerevisiae* [29] and mammalian cells [42], [43], in which MVB formation is impaired. These observations indicate that ORP molecules are required for efficient membrane protein degradation via the MVB sorting pathway. On the other hand, endocytosed soluble proteins, such as GFP and Texas-red BSA, are normally delivered to lysosomes and are efficiently degraded in *obr*s coelomocytes (Figure 3K, 3L, and 3P–3R). This data indicate that, at least in *obr*s coelomocytes, endocytic trafficking from the plasma membrane to lysosomes is not affected and that fusion of late endosomes and lysosomes occurs normally to generate mature lysosomes. Together, these observations suggest that ORP molecules are selectively involved in the degradation of membrane proteins via the MVB sorting pathway. In this study, we analyzed embryonic epithelial cells (Figure 2I–2Q, Figure 5F and 5G) and fertilized eggs (Figure 4) to examine the degradation of membrane cargos (LET-23::GFP and CAV-1::GFP, respectively), and analyzed coelomocytes (Figure 3I–3R) to examine the degradation of lumenal cargos (GFP and Texas-red BSA). The finding that lumenal cargos are normally degraded while membrane cargos are not may be because of tissue differences rather than differences in the cargo-specific functions of ORPs. Therefore, further analyses will be needed to determine if ORPs are involved in the degradation of lumenal cargos in general.

### Sufficient cholesterol is required for MVB formation

How are ORP molecules involved in MVB formation? In the present study, we showed that the total cholesterol content in *obr*s mutants was significantly reduced compared to wild-type worms, indicating that ORPs are important for utilization of cholesterol in *C. elegans* (Figure S11C). We also demonstrated that the cholesterol content of late endosomes/lysosomes was reduced in *obr*s mutants (Figure S11A and S11B). How *C. elegans* ORPs control the intracellular cholesterol level is unclear at this time. As mentioned above, ORPs are implicated in many cellular processes including signal transduction, cholesterol metabolisms, vesicular transport and nonvesicular sterol transport [20]. One possibility is that ORPs is involved in cholesterol transport to late endosomes/lysosomes directly by binding cholesterol or indirectly by regulating other cholesterol-binding proteins. ORPs may also control intracellular signaling and/or vesicular transport that determine the cholesterol content among intracellular organelles.

In *obr*s mutants, knockdown of MVB-related genes remarkably increased embryonic lethality (Figure 1). Knockdown of MVB-related genes also induces high penetrance embryonic lethality under cholesterol-restricted conditions (Figure 5A). Furthermore, late-endosomes/lysosomes are enlarged in both *obr*s mutants and cholesterol-restricted worms (Figure 2B and Figure 5C). These observations suggest that in *obr*s mutants, reduction of late endosomal/lysosomal cholesterol content disturbs MVB formation to some extent, and leads to hypersensitive lethality when the expression of MVB-related genes is knocked down. Another possibility is that the reduced cholesterol content in late endosomes/lysosomes indirectly affects MVB function. For example, the reduced cholesterol content might inhibit Golgi-to-lysosome transport of proteins that are required for MVB formation.

In addition to acting as cholesterol transfer proteins, ORPs have also been proposed to act as a sterol sensor that controls cell signaling [44]. Furthermore, two yeast ORPs (Osh6p and Osh7p) have been shown to interact with Vps4p, which has a role in dissociating the ESCRT-III complex from the endosomal membrane [45], suggesting that ORPs directly regulate ESCRT function in response to the cellular cholesterol content. We found that the localization of an ESCRT-III component (VPS-20) is not affected in *obr*s mutants (Figure S12J, S12K, S12L) and that the localization of mCherry::OBR-2, which fully restores the lysosomal morphology of *obr*s mutants (Figure S12B, S12C, S12D, S12E, S12F), is not altered by knockdown of the MVB-related genes (data not shown). Further studies are needed to determine whether ORPs are directly involved in ESCRT function.

The formation of MVBs is unique in that it is directed toward the lumen of the compartment, rather than the cytosol [46]. During MVB formation, curvature-inducing proteins, such as clathrins and coat protein complexes, could not be involved in the inward invagination of the endosomal membrane. It is also unlikely that the ESCRT proteins directly induce the invagination of the endosomal membrane without getting trapped in the lumen of the forming vesicles. Under these circumstances, lipids have been assumed to play an important role in the membrane invagination step by creating local membrane environments [47]. In mammalian cells, cholesterol is concentrated in endosomal/lysosomal compartments, especially in the luminal vesicles of MVBs [48]. *C. elegans* also has a considerable amount of cholesterol in the endosomal/lysosomal fraction (Figure S11A and S11B). However, the mechanism for accumulation of cholesterol in endosomes/lysosomes is largely unknown, and consequently, the biological significance of cholesterol in endosomal/lysosomal compartments has not been fully elucidated. In this study, we showed that disruption of ORPs reduces the cholesterol content in the endosomal/lysosomal compartments and impairs the MVB formation and function. Although it is not clear at present that the decrease in the cholesterol content is a direct cause of MVB abnormalities, the present study lay a firm basis for further work to more fully elucidate how cholesterol is involved in MVB formation.

In *C. elegans*, cholesterol depletion induces multiple responses such as embryonic lethality, dauer larva formation, and molting defects [38], [39]. Dauer larva formation is regulated by steroid hormone signaling, in which cholesterol-metabolizing enzymes DAF-36 (Rieske-like oxygenase) and DAF-9 (Cytochrome P450) are thought to convert cholesterol into steroid hormones, such as 4-dafachonic acid, that act on a steroid hormone receptor, DAF-12 [49], [50]. *C. elegans* molting is also thought to be regulated by cholesterol-derived steroid hormones via a steroid hormone receptor, NHR-25 [51]. We have never observed dauer larva formation or molting defects in *obr*s mutants, suggesting that *obr* mutations do not affect signaling by these steroid hormones.

### An evolutionarily conserved role of ORPs

In this study, we demonstrated that human ORP1L is required for MVB formation in mammalian cells. A previous study demonstrated that the GTPase Rab7, when bound to GTP, simultaneously binds to ORP1L and RILP to form a RILP-Rab7-ORP1L complex, which is required for the perinuclear localization of late endosomes/lysosomes [52], [53]. Mammalian ORP1L contains three ankyrin repeats at the amino-terminal end, and the interaction with Rab7 through the ankyrin repeats of ORP1L is essential to specify the perinuclear localization of late endosomes/lysosomes (Figure S1) [41]. In *C. elegans* and *D. melanogaster*, the *obr* gene products lack the amino-terminal ankyrin repeats and the late endosomes/lysosomes are not organized into the characteristic perinuclear cluster observed in mammalian cells (Figure S1). These observations suggest that the fundamental role of ORP1L is to maintain enough cholesterol in late endosomes/lysosomes for normal MVB formation. They also suggest that the perinuclear localization of late endosomes/lysosomes in mammals is the result of the appearance of the amino-terminal ankyrin repeats of ORP1L.

As mentioned above, MVB formation requires the inward invagination of the endosomal membrane. Similar membrane invagination also occurs in exosome formation, cytokinesis and viral budding. There is accumulating evidence that the ESCRT proteins have a role in this type of membrane fission. HIV budding from the plasma membrane also requires ESCRT proteins such as Hrs, a homologue of *hgrs-1*. Interestingly, it has been reported that HIV envelopes contain a high level of cholesterol and cholesterol depletion impairs HIV-1 budding at the plasma membrane. Further studies are needed to assess the involvement of ORP proteins in this process.

### Other enhancers whose knockdown causes synthetic lethality with *obr* mutations

In addition to 6 MVB-related genes (*hgrs-1*, *vps-28*, *vps-2*, *vps-20*, *vps-4*, and *vps-34*), we identified 22 other genes that showed synthetic lethality in *obr* quadruple mutants (Table S2). At the present time, the reason for the strong interaction between these 22 genes and *obr* genes is unclear. However, like MVB-related genes, several enhancer genes may require a cholesterol-rich membrane environment for their normal functions. Cholesterol-rich microdomains play important roles in several biological functions, such as raft-dependent cellular signaling and caveolae-mediated endocytosis at the plasma membrane [15]. The present study suggested a novel role of cholesterol-rich microdomains, i.e. providing an adequate membrane environment for MVB formation. Further studies of the enhancer genes should uncover other aspects of intracellular cholesterol functions.

## Materials and Methods

### *C. elegans* strains and methods

Worm cultures, genetic crosses, and other *C. elegans* methods were performed according to standard protocols [54] except where otherwise indicated. *obr-1(xh16)*, *obr-2(xh17)*, *obr-3(tm1087)* and *obr-4(tm1567)* mutants were isolated by TMP (trimethylpsoralen)/UV method [26] and were backcrossed onto the wild-type background five times before phenotypic analysis. Transgenic strains used for this study are *cdIs36[punc-122p::C31E10.7::GFP]* for endoplasmic reticulum, *cdIs54[pcc1::MANS::GFP]* for Golgi, *pwIs50[lmp-1::GFP]* for lysosomes, *cdIs85[pcc1::2xFYVE::GFP]* for early endosomes, *bIs34[rme-8::GFP]* for late endosomes, *cdIs39[pcc1::GFP::RME-1]* for recycling endosomes, *arIs37[myo-3p::ssGFP]*, *pwIs28[pie-1p-cav-1::GFP7] tmIs105[vit-2::GFP]*, *xhIs2501[dpy-7p::let-23::GFP]*, *xhEx2503[obr-2 genome::GFP]*, *xhEx2511[unc122p::mCherry::obr-1]*, *xhEx2512[unc122p::mCherry::obr-2]*, *xhEx2513[unc122p::mCherry::obr-3]*, and *xhEx2514[unc122p::mCherry::obr-4]*. Some of the strains used in this work were obtained from *Caenorhabditis* Genetics Center, University of Minnesota, Minneapolis, MN).

### Phenotypic analysis

Adult wild-type and mutant worms were allowed to lay eggs for 2–3 hr, and the progeny were scored for embryonic lethality and larval arrest. Unhatched eggs were examined 24 hr after being laid, and hatched but arrested larvae were examined 72 hr after being laid. To perform fluid-phase endocytosis assay, Texas red BSA was injected at 1 mg/ml in water into the body cavity of wild-type or *obr* quadruple mutants expressing RME-8::GFP. At defined time points, animals were mounted on slides, put on ice to stop endocytosis, and fluid-phase internalization of the dye into the coelomocytes was viewed with a confocal microscope. For the quantification of endosomes and lysosomes sizes, discrete intracellular structures in at least 30 coelomocytes were analyzed for each marker (RME-8::GFP for late endosomes, LMP-1::GFP for lysosomes). Individual sections through coelomocyte were scanned, and the diameter of the largest endosomes or lysosomes was scored. Coelomocyte, endosomes and lysosomes areas were calculated from their diameter. To quantify the size of LET-23::GFP-positive endocytic compartments in embryos, LET-23::GFP-positive endocytic compartments were sorted into three size categories according to their diameter: 0.7µm>(normal), 0.7–1.5 µm (weak enlarged), and 1.5 µm<(strong enlarged).

### RNAi feeding screen

Feeding RNAi was performed as described previously [55]. To score embryonic lethality, young adult worms were placed on each RNAi plate and allowed to feed for 24 hr. Three worms from the original plate were transferred to a fresh RNAi plate and were allowed to lay eggs for 4–5 hr to score embryonic lethality. In an RNAi screen, we first used feeding RNAi clones on chromosome I and III in the Ahringer library to identify RNAi clones that cause high penetrance embryonic lethality in the *obr* quadruple mutant background, but not in the wild-type background. As a result, we found 22 RNAi clones that caused synthetic lethality with *obr* quadruple mutations (Table S2, Group A). These enhancer genes included the genes encoding vesicular transport-related proteins, such as *apm-1* (μ subunit of AP-1), *arf-1.2* (a homologue of ARF), *vps-34* (Class III phosphatidylinositol 3 kinase) and *vps-2* (ESCRT III). Therefore we next focused on genes whose homologues are known to regulate intracellular vesicular transport in other species (Table S2) [56] (MVB formation-related genes, small G proteins, components of COG complex, SNARE genes, SEC-1 family genes, coatmer proteins, and components of retromer complex). We tested 113 genes listed in Table S1 and identified another six genes that could enhance embryonic lethality of *obr* quadruple mutants (Table S2, Group B).

### Cholesterol depleted condition in *C. elegans*

To obtain cholesterol-free conditions, agar was replaced by agarose S (Wako, Japan) and peptone was omitted from plates. An overnight culture of the OP50 strain of *E. coli* was grown on a LB medium. Bacteria were rinsed with M9 medium before use. Bacterial suspension were spread on cholesterol-free agarose plates. To perform RNAi under cholesterol depleted condition, bacteria were grown at 37°C to an O.D. of 0.5–0.8, induced with 0.4mM IPTG for 4hr, then concentrated and spread onto agarose plates containing 0.4mM IPTG. For feeding P0 animals, L4 hermaphrodites were plated directly on these plates at 20°C and their progeny were analyzed.

### Microscopy

Fluorescence images were obtained using an Axio Imager M1 (Carl Zeiss MicroImaging Inc., Japan) microscope equipped with a digital CCD camera. Confocal images were obtained using a Zeiss LSM510 META confocal microscope system (Carl Zeiss MicroImaging).

### Cell culture and transfection

HeLa cells were grown in DMEM, 10% fetal bovine serum (FBS), 100 U/ml penicillin, 100 mg/ml streptomycin, and 2mM L-glutamine. Cells were transiently transfected for 24–36 hr with cDNA constructs in complete medium using LipofectAMINE 2000 (Invitrogen, San Diego, CA, USA). Transfections were carried out according to the manufacturers' instructions. To perform RNAi, the cells were transfected for 48 hr with 20nM ORP1L-specific (sense strand GGACGAAAGGAGUUGGUAAdTdG) or control siRNA (Nippon EGT, Japan) using Lipofectamine 2000 (Invitrogen, San Diego, CA, USA).

### Preparation of polyclonal antibody

A glutathione S-transferase–ORP1L fusion protein corresponding to amino acids 428–553 in the ORP1L protein was expressed in *E. coli* BL21 (DE3), purified by affinity chromatography on glutathione-Sepharose 4B (Pharmacia AB, Uppsala, Sweden), and used for immunization of New Zealand White rabbits according to a standard protocol. The ORP1L antiserum were purified by using an Affi-Gel (BIO-RAD, Japan) to which the antigen fragment had been coupled. The antibody were used for immunoblotting in 1 ∶ 10 dilution.

### Electron microscopy

HeLa cells cultured on plastic cover glass (Celldesk LF1, Sumitomo Bakelite inc, Tokyo, Japan) in 24-well culture plates were fixed with 2.5% glutaraldehyde in 0.1 M phosphate buffer (pH 7.4) for 2 hr. Cells were post-fixed in 1% OsO_4_ in the same buffer for 1 hr, and dehydrated with a series of ethanol and embedded in epon. After the resin hardened, Celldesk was removed from the epon block. Ultra-thin sections were cut horizontally to the bottom of Celldesk, stained with uranyl acetate for 60 minutes, stained with lead citrate solution for 1 min, and observed under a Hitachi H-7600 electron microscope. For quantitative analyses, electron micrographs were taken at a magnification of 12,000. The cytoplasmic area and the number and diameter of MVBs were determined. Ten cell profiles were taken from each Celldesk, and three samples were analyzed (a total of 30 cells). *C. elegans* were pre-fixed with 4% paraformaldehyde and 1% glutaraldehyde in 0.1 M phosphate buffer (pH 7.4). Samples were then cut into small pieces, fixed again with 2% paraformaldehyde and 2% glutaraldehyde in the same buffer, and post-fixed with 2% osmium tetroxide in phosphate buffer for 4 hrs. Afterwards, fixed specimens were dehydrated in a graded series of ethanol and embedded in Quetol 651 epoxy resin. Ultrathin (80 to 90 nm-thick) sections obtained by ultramicrotomy were stained with uranyl acetate for 15 minutes and with modified Sato's lead solution for 5 mins. TEM observation was performed using a JEOL JEM-1200EX electron microscope.

### Measurement of cholesterol level in late endosomes/lysosomes in *C. elegans*

Synchronized first-stage larvae (40,000 worms) were cultured with 6 µCi of [^14^C]-cholesterol (54 mCi/mmol; American Radiolabeled Chemicals, Inc. St. Louis, U.S.A.) for 54 hr on cholesterol free agar plates (see above) and were harvested from the plates with M9 medium. Late endosomal/lysosomal fraction was then prepared using the lysosome isolation kit (Sigma). Briefly, worms were homogenized using a Dounce homogenizer device and the lysates were subjected to centrifugation at 1,000×*g* to remove the nuclei. The post nuclear supernatant was subjected to centrifugation at 20,000×*g* to pellet the membranes, yielding the crude membrane fraction. The crude membrane fraction was resuspended in extraction buffer and subjected to density gradient ultracentrifugation at 150,000×*g* on an 8–27% Optiprep gradient for 4 hr (Lysosomal Isolation Kit, Sigma-Aldrich). 250 µl fractions were collected from the bottom of the tube with a peristaltic pump. The resulting fraction was treated with 250 mM calcium chloride to remove residual mitochondria and rough ER. Aliquots were assayed for lipid analysis, and the remaining material was processed for immunoblotting. Lipids were extracted by hexane, and were separated by one-dimensional TLC on silica gel 60 plates (Merck Biosciences) in chloroform-methanol (24∶1). Cholesterol was identified by comigration with known standard. Cholesterol ratio of late endosome/lysosomal fraction (fraction 7 and 8) was expressed as the percentage of radioactivity of 20,000×*g* ppt.

## Supporting Information

Figure S1 ORP Family in *H. sapiens*, *C. elegans*, and *S. cerevisiae*. The *H. sapiens*, *C. elegans*, and *S. cerevisiae* ORP families. Domain structures of the major variants are shown. The human proteins can be subdivided into six subfamilies (indicated with Roman numerals) based on gene structure and amino acid homology. In *C. elegans*, 4 ORP members are conserved (OBR-1, OBR-2, OBR-3, OBR-4) and classified into the subfamilies I, II, IV, and V, respectively. Yeast ORP members (OSH1 to Osh7) share comparatively low sequence homologies with mammalian ORP proteins. Blue box, PH domain; red box, sterol binding domain; yellow box, EQVSHHPP motif which is fully conserved in all members of the family; green box, hydrophobic region; tangerine box, ankyrin-repeat; pink box, Golgi dynamics domain.
(0.26 MB TIF)

Figure S2 Gene structures of *obr-1*, *obr-2*, *obr-3* and *obr-4*. Genomic structures of *obr-1*, *obr-2*, *obr-3* and *obr-4*. Boxes represent exons. The start (ATG) and stop (TAG or TGA) codons are indicated above the first and last exons of each gene. The EQVSHHPP motif, which is completely conserved in all ORP family proteins, is indicated in yellow. Red, blue, and green indicate the regions encoding the sterol-binding domain, PH domain, and hydrophobic putative transmembrane domain. The extent of the deletion in *obr-1(xh16)*, *obr-2(xh17)*, *obr-3(tm1087)*, and *obr-4(tm1567)* is indicated by a horizontal line. *obr-1(xh16)* and *obr-3(tm1087)* contain 1716-bp and 613-bp deletions, respectively, in their sterol-binding domains. *obr-1(xh16)* allele lacks an ORP signature “EQVSHHPP” motif. *obr-3(tm1087)* harbors an in-frame deletion located 125 amino acids downstream of its “EQVSHHPP” motif and lacks 22 amino acids in the sterol-binding domain. *obr-2(xh17)* is a 1724 bp deletion and removes the N-terminal half of the protein including its ATG initiation codon. *obr-4(tm1567)* possesses a 540-bp deletion which causes a premature stop codon, resulting in a truncated protein lacking the sterol-binding domain.
(0.36 MB TIF)

Figure S3 Structure of subfamily I ORP proteins. Multiple sequence alignment of the conserved sterol-binding domain of the *C. elegans* OBR-1 and homologous sequences in *D. melanogaster* (dobr-1), human (hOSBP, hORP4), and mouse (mOSBP, mORP4). Sequences were aligned with Clustal W. Residues identical, or related, in three or more of the sequences are indicated by black or gray boxes, respectively. The number on the right indicates amino acid positions. The EQVSHHPP motif is underlined in yellow. Accession numbers for the sequences used were as follows: *C. elegans* OBR-1: NP_499448; *D. melanogaster* OBR-1: NP_477271; human OSBP: NP_002547; mouse OSBP: NP_001028346; human ORP4: NP_110385; mouse ORP4: NP_690031.
(3.35 MB TIF)

Figure S4 Structure of subfamily II ORP proteins. Multiple sequence alignment of the conserved sterol-binding domain of the *C. elegans* OBR-2 and homologous sequences in *D. melanogaster* (dobr-2), human (hORP1L, hORP2), and mouse (mORP1L, mORP2). The EQVSHHPP motif is underlined in yellow. Accession numbers for the sequences used were as follows: *C. elegans* OBR-2: NP_506695; *D. melanogaster* OBR-2: NP_611865; human ORP1L: NP_542164; mouse ORP1L: NP_997413; human ORP2: NP_653081; mouse ORP2: NP_653083.
(3.97 MB TIF)

Figure S5 Structure of subfamily IV ORP proteins. Multiple sequence alignment of the conserved sterol-binding domain of the *C. elegans* OBR-3 and homologous sequences in *D. melanogaster* (dobr-3), human (hORP5, hORP8), and mouse (mORP5, mORP8). The EQVSHHPP motif is underlined in yellow. Accession numbers for the sequences used were as follows: *C. elegans* OBR-3: NP_741923; *D. melanogaster* OBR-3: NP_650878; human ORP5: NP_065947; mouse ORP5: NP_077251; human ORP8: NP_065892; mouse ORP8: NP_780698.
(3.62 MB TIF)

Figure S6 Structure of subfamily V ORP proteins. Multiple sequence alignment of the conserved sterol-binding domain of the *C. elegans* OBR-4 and homologous sequences in *D. melanogaster* (dobr-4), human (hORP9), and mouse (mORP9). The EQVSHHPP motif is underlined in yellow. Accession numbers for the sequences used were as follows: *C. elegans* OBR-4: NP_491691; *D. melanogaster* OBR-4: NP_610534; human ORP9: NP_078862; mouse ORP9: NP_598646.
(2.54 MB TIF)

Figure S7 *obr* quadruple mutants exhibit abnormal hypodrmis and cuticle. Transmission electron micrographs of wild-type (A and C) and *obr* quadruple mutants (B and D). (A–D) Transverse sections through the cuticle. In wild-type worms, the three ridges of the alae are observed (A, arrowheads), and the cuticle is approximately 0.5 µm in thickness with a flat surface (C, arrowheads). On the other hand, in *obr* quadruple mutants (*obr-1;2;3;4*), the morphology of alae is severely affected (B, arrows), the cuticle's outer surface is wavy instead of flat (D, arrows). Note that *obr* quadruple mutants have enlarged vacuoles which are not observed in wild-type worms (B, asterisks). Scale bar represents 2 µm.
(3.42 MB TIF)

Figure S8 *obr* quadruple mutants exhibit no abberation with receptor-mediated endocytosis. (A–D) Fluorescence images and the corresponding Nomarski micrographs of adult hermaphrodites of wild-type and *obr* quadruple mutants carrying the YP170::EGFP transgene. The YP170::EGFP fusion protein is transported like endogenous yolk, from intestine to oocyte via receptor-mediated endocytosis. In wild-type worms, the YP170::EGFP endocytosed two nearly full-grown oocytes of one gonad arm (A, arrows). In *obr* quadruple mutants (*obr-1;2;3;4*), YP170::EGFP is efficiently endocytosed and stored in oocytes in a similar manner to that in wild-type worms (C, arrows). Scale bars are 20 µm.
(0.49 MB TIF)

Figure S9 Morphology of ER, Golgi, and early endosomes is not affected in *obr* quadruple mutants. Confocal micrographs of wild-type and *obr* quadruple mutant coelomocytes (*obr-1;2;3;4*) expressing a GFP fusion organelle marker. TRAM; rER marker, AMAN-2 (mannosidase II); Golgi marker, 2xFYVE; early endosomal marker. The outline of the coelomocyte is indicated by a white line. All scale bars are 5 µm. (G) Quantification of the size of early endosomes in wild-type, *obr* quadruple mutants and *vps-4* (RNAi) coelomocytes. The vertical axis indicates the ratio of early endosomal area per coelomocyte area.
(0.72 MB TIF)

Figure S10 CAV-1::GFP is degraded after fertilization. (A and B) Normarski (A) and fluorescence (B) micrographs of wild-type hermaphrodites expressing CAV-1::GFP. In the proximal gonad, oocytes undergo maturation (A, arrowheads) and are ovulated into the sperm-containing spermatheca (A, asterisk) where they are fertilized. Fertilized eggs then move into the uterus (A, arrows). In control oocytes prior to fertilization, CAV-1::GFP is concentrated in intracellular vesicles and large ring-like cytoplasmic structures and localized weakly to the plasma membrane (A and B, arrowheads). Immediately after oocytes pass through the spermatheca and are fertilized, the amount of CAV-1::GFP on the cell surface rapidly increases, followed by its internalization and degradation. Newly fertilized embryos exhibited bright CAV-1::GFP fluorescence, initially at the cell surface (A and B, red arrows) and subsequently on internal membranes, but embryos beyond the 2-cell stage, approximately 90 minutes post fertilization, lacked visible fluorescence (A and B, white arrows).
(0.26 MB TIF)

Figure S11 Late endosomal/lysosomal cholesterol is reduced in *obr* quadruple mutants. (A) Wild-type and *obr* quadruple mutants (*obr-1;2;3;4*) were disrupted with a Dounce homogenizer and the membrane fractions (20,000*g* ppt) were subjected to continuous OptiPrep density-gradient centrifugation (for details, see Materials and Methods). Aliquots of 1,000*g* sup, 20,000*g* sup, 20,000*g* ppt, and gradient fractions 1–8 were analyzed by immunoblotting using antibodies against the indicated proteins [1],[2]. The late endosomal/lysosomal fractions of worms were found at fractions 7 and 8. Lipids of each fraction were extracted and analyzed by TLC. The band corresponding to cholesterol was measured. (B) The amount of cholesterol in each fraction was quantified by densitometry and expressed as the percentage of cholesterol content of 20,000 *g* ppt. Similar data showing reduced cholesterol content in late endosomal/lysosomal fractions were obtained from two independent experiments. (C) Total cholesterol content in wild-type and *obr* quadruple mutants. Cholesterol amounts are expressed as nanomoles of cholesterol per nanomole of phospholipids.
(0.57 MB TIF)

Figure S12 LET-23::GFP localized in enlarged endosomes/lysosomes.(A) Synergism between *obr* genes and *vps-4*. Diameter of LysoSensor-positive vesicles is classified into three categories: Normal (normal: <1.5 µm), Weak (weakly enlarged vesicle: 1.5–2 µm) and Strong (strongly enlarged: >2 µm). Graph shows the percentage of worms containing each category of LysoSensor-positive vesicles. (B–F) Expression of mCherry::OBR-2 fully rescues the enlarged late-endosomes/lysosomes in coelomocytes of *obr* quadruple mutants. (B–F) Confocal micrographs of coelomocytes expressing LMP-1::GFP. Wild-type (B), *obr* quadruple mutants (C), and *obr* quadruple mutants expressing mCherry::OBR-2 under the control of coelomocyte-specific *unc-122* promoter (D). An arrow indicates abnormally enlarged lysosomes, and arrowheads indicate normal lysosomes. (E, F) Subcellular localization of OBR-2. mCherry::OBR-2 mainly localized in the cytosol. Note that expression of mCherry::OBR-2 fully rescues the enlarged lysosomes in *obr* quadruple mutant coelomocyes. The outlines of the coelomocytes are indicated by a white line. (G–I) *obr* quadruple mutants accumulate LET-23::GFP, a *C. elegans* EGF receptor. Confocal micrographs of embryos in *obr* quadruple mutants carrying the LET-23::GFP transgene *[dpy-7p::let-23cDNA::GFP]*. The transgenic worms were grown on plates containing Lysotracker red. The area enclosed by the white line indicates the epithelial cells which express LET-23::GFP. (H) Lysotracker red-positive vesicles out of the enclosed line are lysosomes in the cells which do not express LET-23::GFP (mainly intestinal cells and muscle cells). Note that most of the enlarged LET-23::GFP-positive vesicles are stained with Lysotracker red (I, arrows). (J–L) Confocal fluorescence images of wild-type (J), *vps-4* (RNAi) (K), and *obr* quadruple mutants expressing VENUS::VPS-20 (L). The outline of the coelomocyte is indicated by a white line. In wild-type worms, VENUS::VPS-20 is localized in the cytosol. In contrast, VENUS::VPS-20 is translocated from the cytosol to the membrane-like structure, possibly enlarged lysosomes. In *obr* quadruple mutants, VENUS::VPS-20 is localized in the cytosol in a similar manner to that in wild-type worms.
(1.74 MB TIF)

Figure S13 Depletion of ORP1L delays EGF receptor degradation. (A) HeLa cells (control RNAi or ORP1L RNAi) were treated with EGF (100 ng/ml) at 37°C for the periods indicated and the lysates were subjected to Western blot analysis with an anti-EGF receptor antibody. (B) The remaining EGF receptor bands at each time point were quantitated and indicated as a percentage relative to that at time 0 hr.
(0.22 MB TIF)

Table S1 A list of genes tested for synthetic lethality with *obr* quadruple mutations.
(0.13 MB DOC)

Table S2 Genetic enhancers of *obr* quadruple mutants.
(0.08 MB DOC)

Text S1 Supporting materials.
(0.06 MB DOC)
