# Supplementary figures and images for: Multivesicular Body Formation Requires OSBP–Related Proteins and Cholesterol
Source: PLoS Genet. 2010 Aug 5;6(8):e1001055. doi: 10.1371/journal.pgen.1001055 (PMC2916882; doi:10.1371/journal.pgen.1001055)

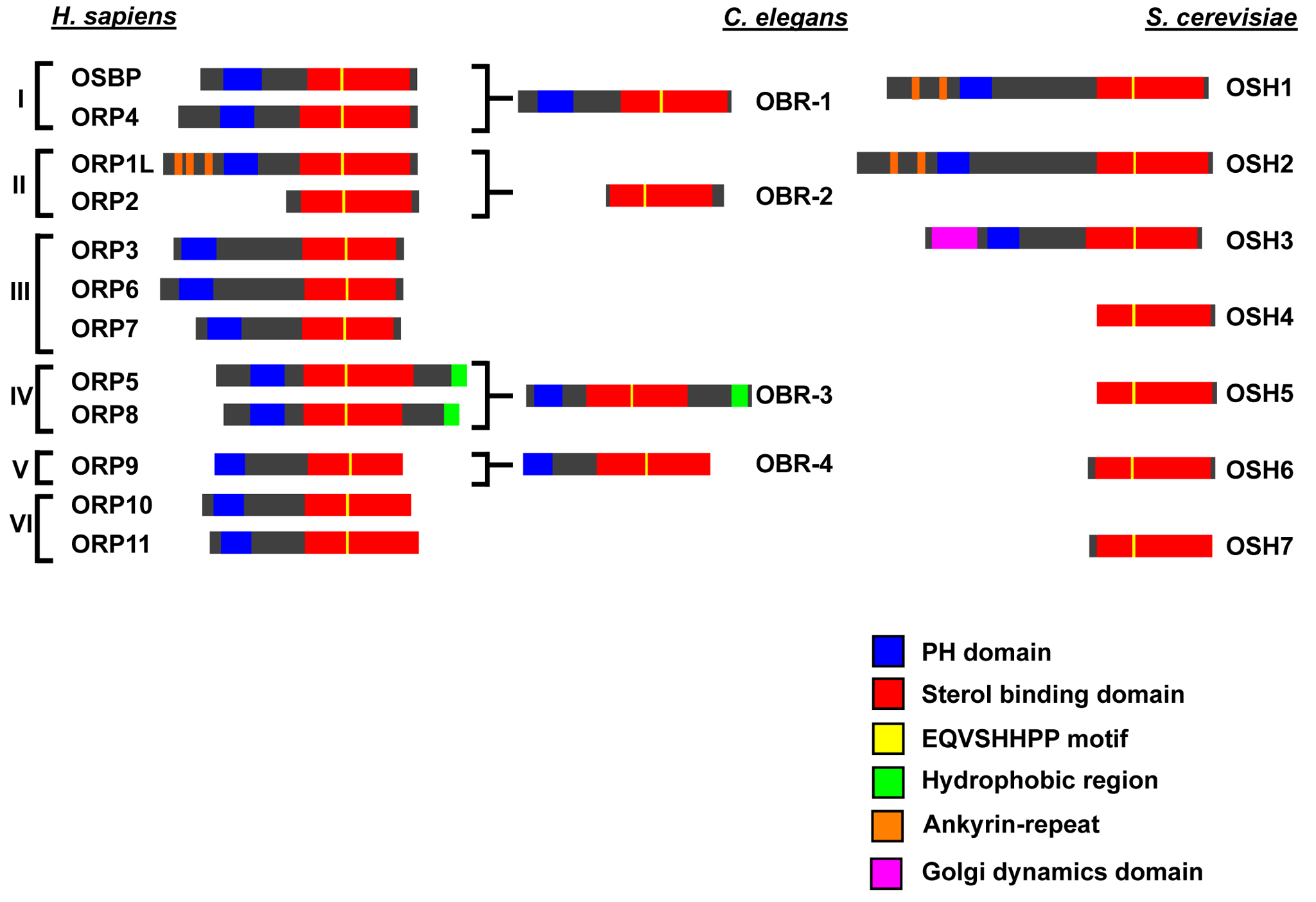

Supplement: Figure S1 — ORP Family in H. sapiens, C. elegans, and S. cerevisiae. The H. sapiens, C. elegans, and S. cerevisiae ORP families. Domain structures of the major variants are shown. The human proteins can be subdivided into six subfamilies (indicated with Roman numerals) based on gene structure and amino acid homology. In C. elegans, 4 ORP members are conserved (OBR-1, OBR-2, OBR-3, OBR-4) and classified into the subfamilies I, II, IV, and V, respectively. Yeast ORP members (OSH1 to Osh7) share comparatively low sequence homologies with mammalian ORP proteins. Blue box, PH domain; red box, sterol binding domain; yellow box, EQVSHHPP motif which is fully conserved in all members of the family; green box, hydrophobic region; tangerine box, ankyrin-repeat; pink box, Golgi dynamics domain. (0.26 MB TIF) [file pgen.1001055.s001.tif]

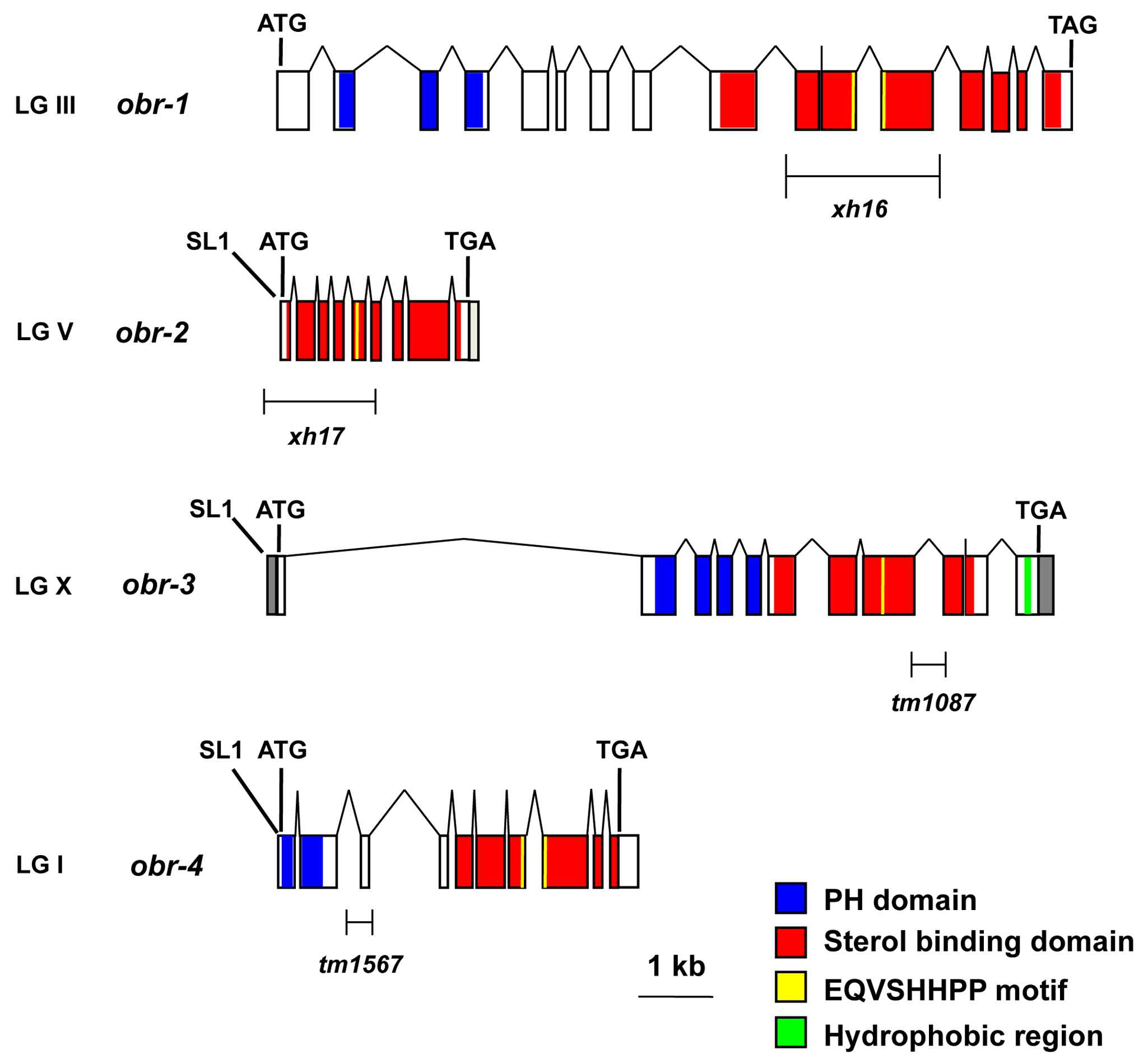

Supplement: Figure S2 — Gene structures of obr-1, obr-2, obr-3 and obr-4. Genomic structures of obr-1, obr-2, obr-3 and obr-4. Boxes represent exons. The start (ATG) and stop (TAG or TGA) codons are indicated above the first and last exons of each gene. The EQVSHHPP motif, which is completely conserved in all ORP family proteins, is indicated in yellow. Red, blue, and green indicate the regions encoding the sterol-binding domain, PH domain, and hydrophobic putative transmembrane domain. The extent of the deletion in obr-1(xh16), obr-2(xh17), obr-3(tm1087), and obr-4(tm1567) is indicated by a horizontal line. obr-1(xh16) and obr-3(tm1087) contain 1716-bp and 613-bp deletions, respectively, in their sterol-binding domains. obr-1(xh16) allele lacks an ORP signature “EQVSHHPP” motif. obr-3(tm1087) harbors an in-frame deletion located 125 amino acids downstream of its “EQVSHHPP” motif and lacks 22 amino acids in the sterol-binding domain. obr-2(xh17) is a 1724 bp deletion and removes the N-terminal half of the protein including its ATG initiation codon. obr-4(tm1567) possesses a 540-bp deletion which causes a premature stop codon, resulting in a truncated protein lacking the sterol-binding domain. (0.36 MB TIF) [file pgen.1001055.s002.tif]

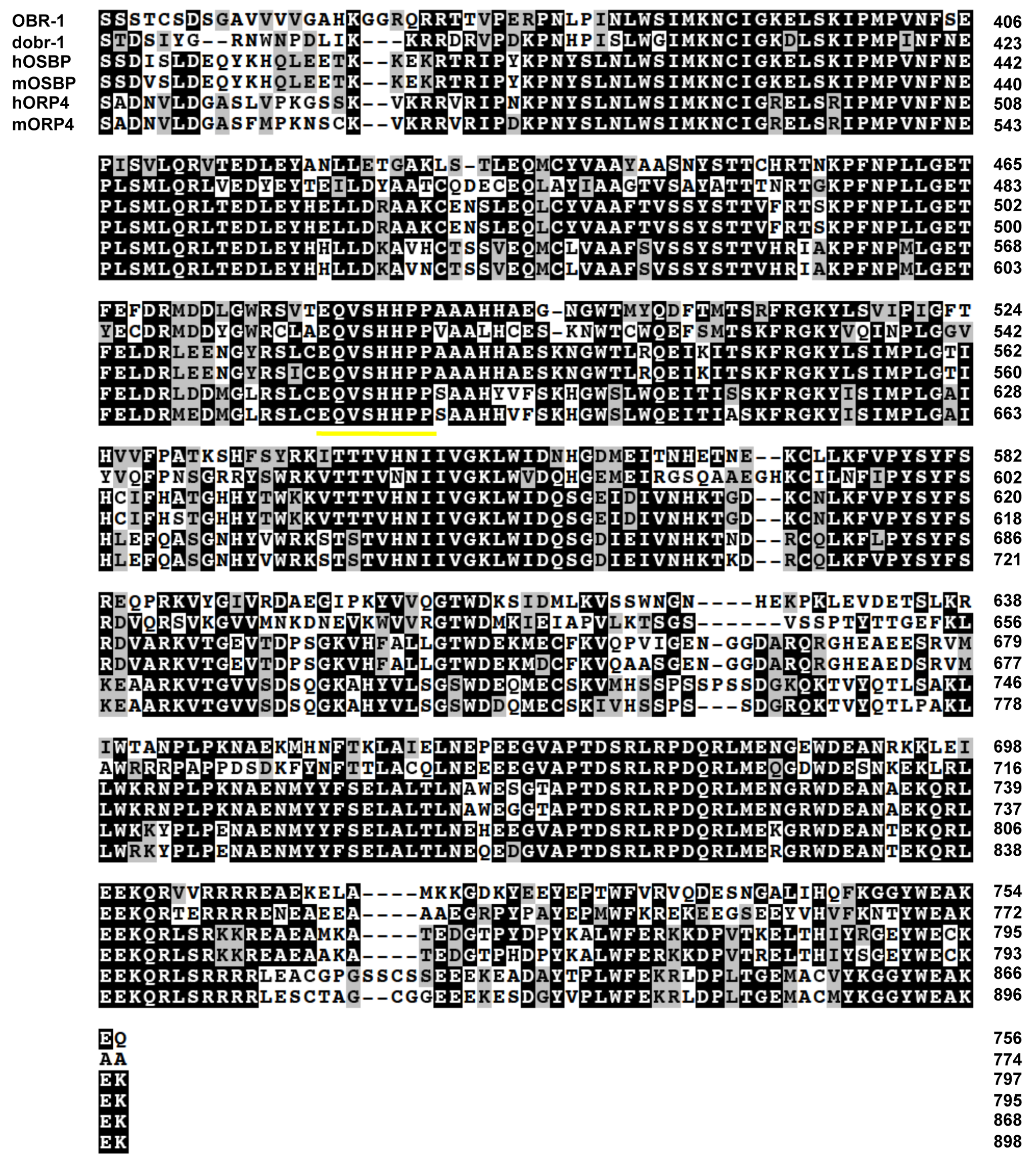

Supplement: Figure S3 — Structure of subfamily I ORP proteins. Multiple sequence alignment of the conserved sterol-binding domain of the C. elegans OBR-1 and homologous sequences in D. melanogaster (dobr-1), human (hOSBP, hORP4), and mouse (mOSBP, mORP4). Sequences were aligned with Clustal W. Residues identical, or related, in three or more of the sequences are indicated by black or gray boxes, respectively. The number on the right indicates amino acid positions. The EQVSHHPP motif is underlined in yellow. Accession numbers for the sequences used were as follows: C. elegans OBR-1: NP_499448; D. melanogaster OBR-1: NP_477271; human OSBP: NP_002547; mouse OSBP: NP_001028346; human ORP4: NP_110385; mouse ORP4: NP_690031. (3.35 MB TIF) [file pgen.1001055.s003.tif]

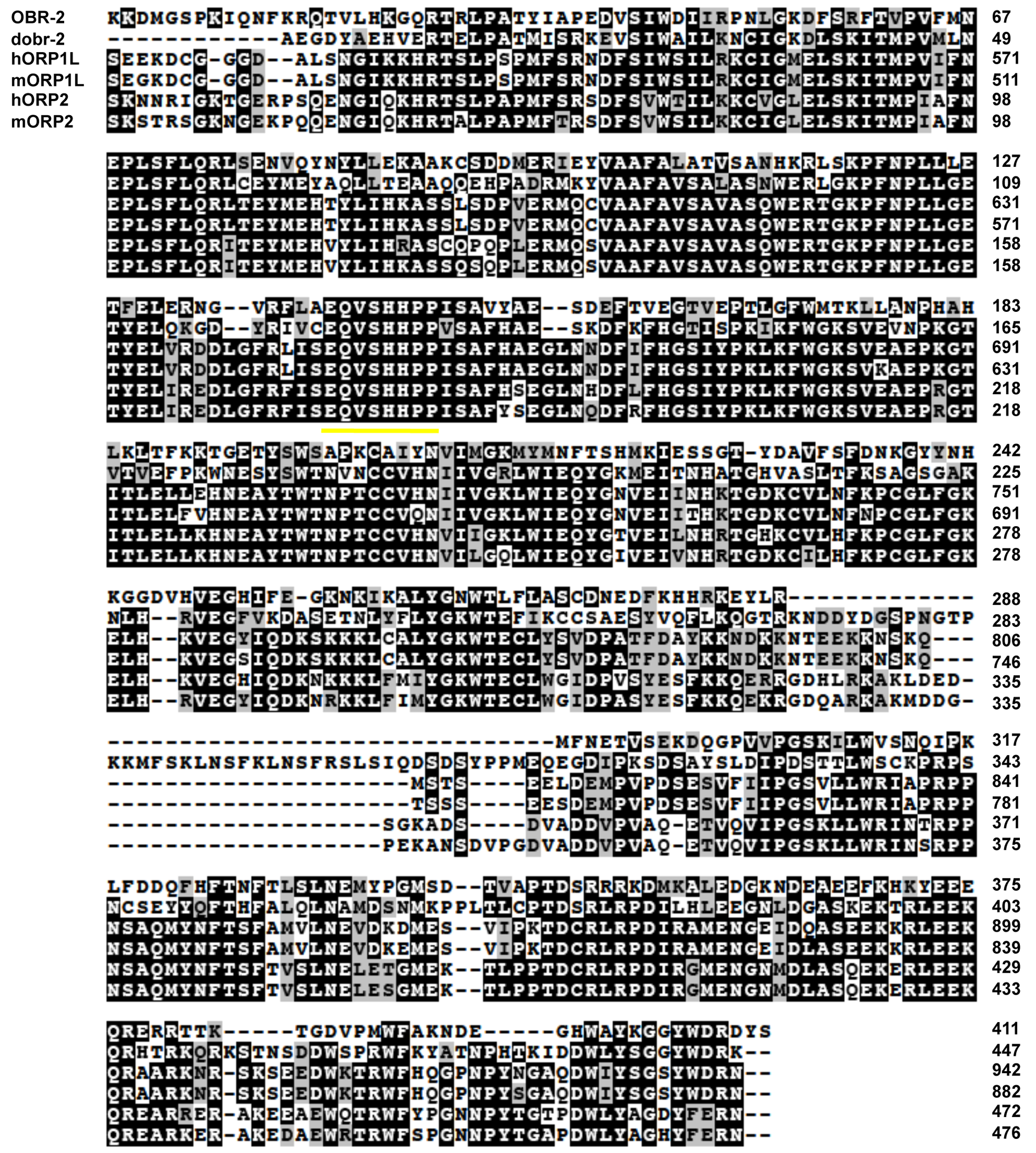

Supplement: Figure S4 — Structure of subfamily II ORP proteins. Multiple sequence alignment of the conserved sterol-binding domain of the C. elegans OBR-2 and homologous sequences in D. melanogaster (dobr-2), human (hORP1L, hORP2), and mouse (mORP1L, mORP2). The EQVSHHPP motif is underlined in yellow. Accession numbers for the sequences used were as follows: C. elegans OBR-2: NP_506695; D. melanogaster OBR-2: NP_611865; human ORP1L: NP_542164; mouse ORP1L: NP_997413; human ORP2: NP_653081; mouse ORP2: NP_653083. (3.97 MB TIF) [file pgen.1001055.s004.tif]

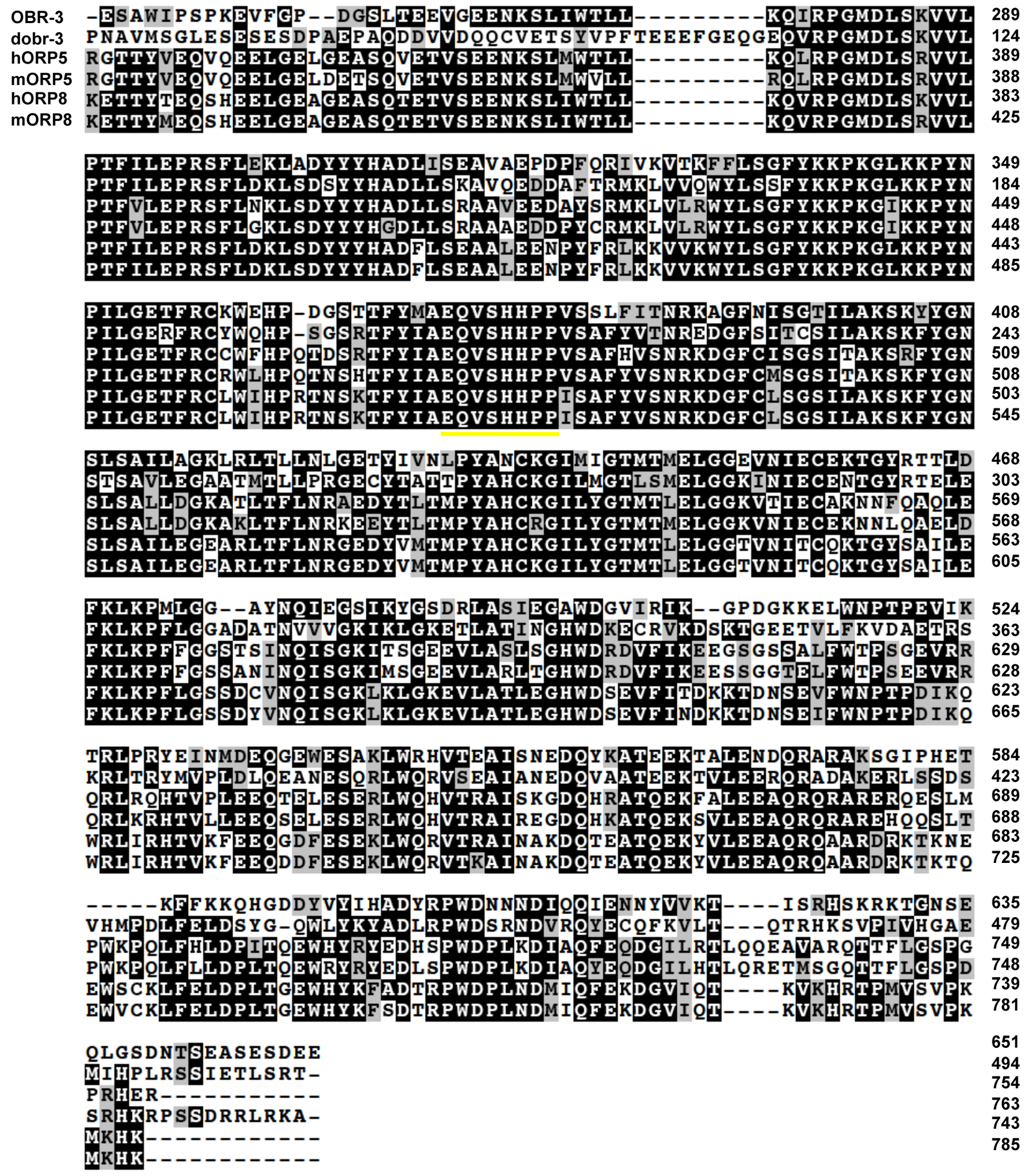

Supplement: Figure S5 — Structure of subfamily IV ORP proteins. Multiple sequence alignment of the conserved sterol-binding domain of the C. elegans OBR-3 and homologous sequences in D. melanogaster (dobr-3), human (hORP5, hORP8), and mouse (mORP5, mORP8). The EQVSHHPP motif is underlined in yellow. Accession numbers for the sequences used were as follows: C. elegans OBR-3: NP_741923; D. melanogaster OBR-3: NP_650878; human ORP5: NP_065947; mouse ORP5: NP_077251; human ORP8: NP_065892; mouse ORP8: NP_780698. (3.62 MB TIF) [file pgen.1001055.s005.tif]

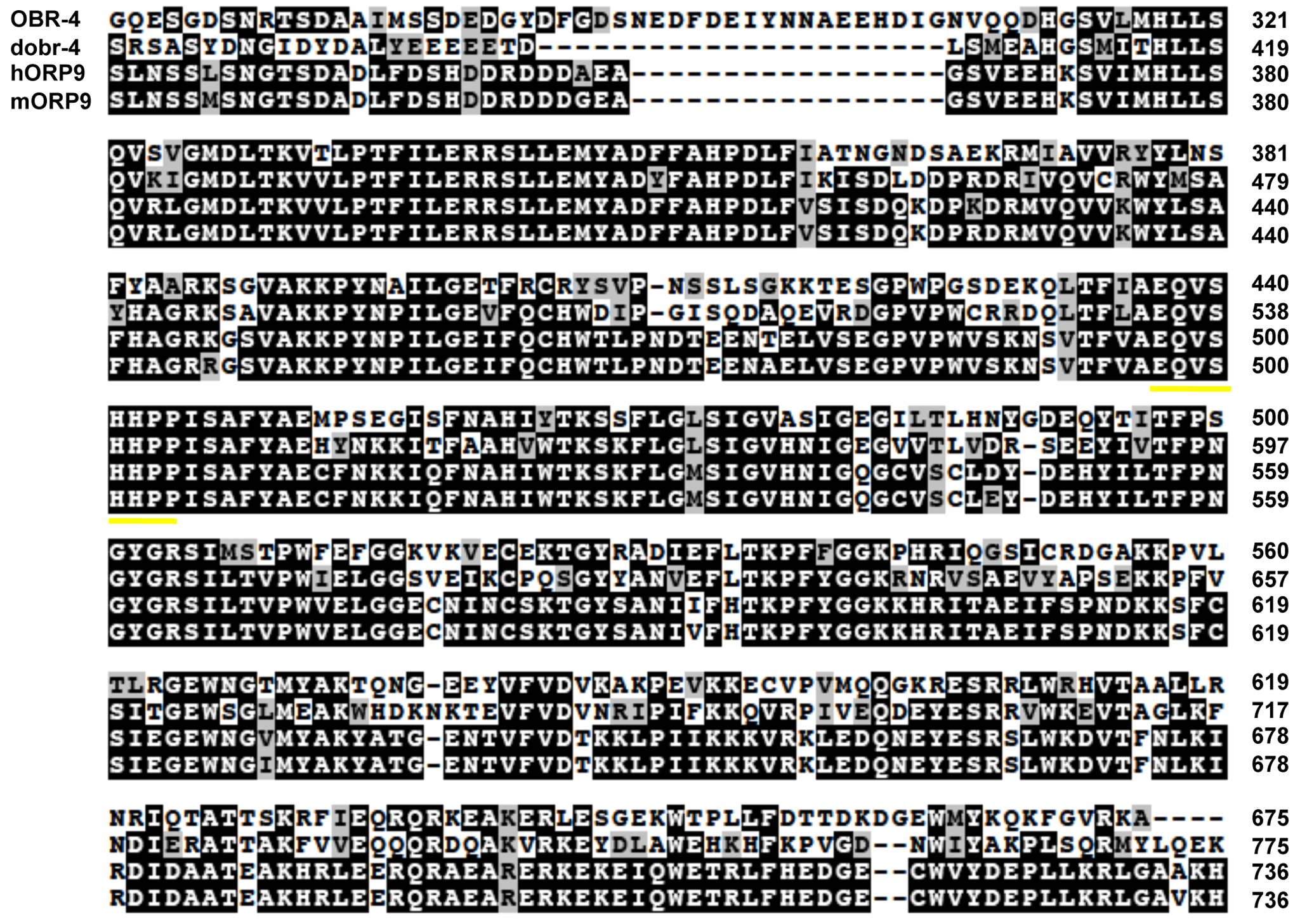

Supplement: Figure S6 — Structure of subfamily V ORP proteins. Multiple sequence alignment of the conserved sterol-binding domain of the C. elegans OBR-4 and homologous sequences in D. melanogaster (dobr-4), human (hORP9), and mouse (mORP9). The EQVSHHPP motif is underlined in yellow. Accession numbers for the sequences used were as follows: C. elegans OBR-4: NP_491691; D. melanogaster OBR-4: NP_610534; human ORP9: NP_078862; mouse ORP9: NP_598646. (2.54 MB TIF) [file pgen.1001055.s006.tif]

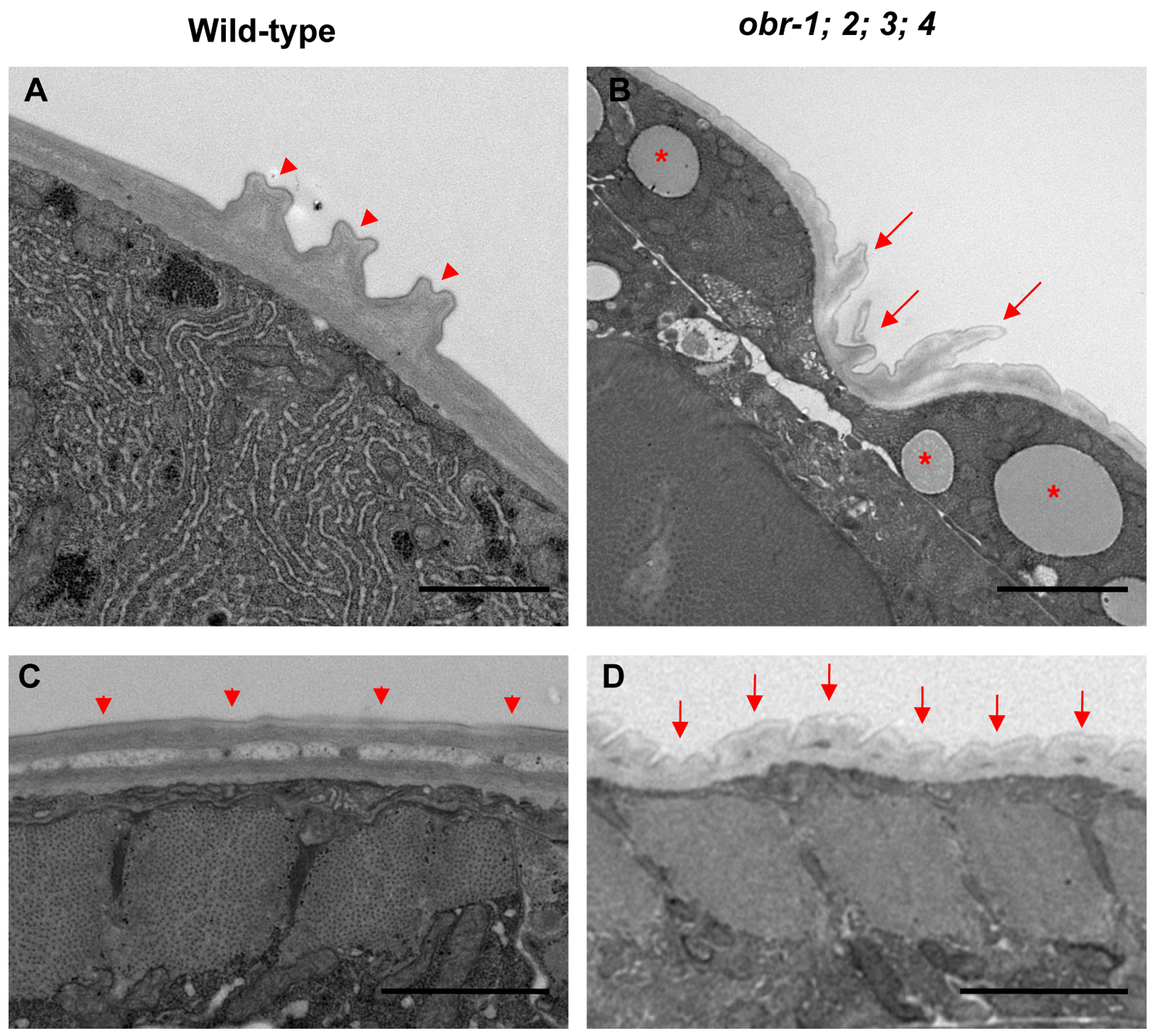

Supplement: Figure S7 — obr quadruple mutants exhibit abnormal hypodrmis and cuticle. Transmission electron micrographs of wild-type (A and C) and obr quadruple mutants (B and D). (A–D) Transverse sections through the cuticle. In wild-type worms, the three ridges of the alae are observed (A, arrowheads), and the cuticle is approximately 0.5 µm in thickness with a flat surface (C, arrowheads). On the other hand, in obr quadruple mutants (obr-1;2;3;4), the morphology of alae is severely affected (B, arrows), the cuticle's outer surface is wavy instead of flat (D, arrows). Note that obr quadruple mutants have enlarged vacuoles which are not observed in wild-type worms (B, asterisks). Scale bar represents 2 µm. (3.42 MB TIF) [file pgen.1001055.s007.tif]

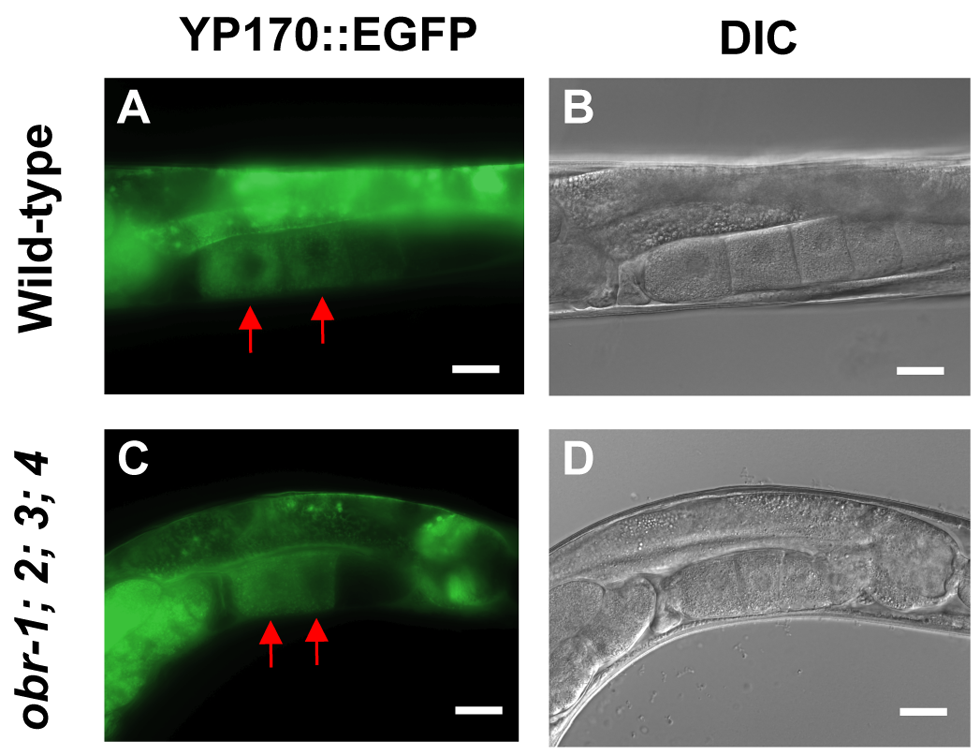

Supplement: Figure S8 — obr quadruple mutants exhibit no abberation with receptor-mediated endocytosis. (A–D) Fluorescence images and the corresponding Nomarski micrographs of adult hermaphrodites of wild-type and obr quadruple mutants carrying the YP170::EGFP transgene. The YP170::EGFP fusion protein is transported like endogenous yolk, from intestine to oocyte via receptor-mediated endocytosis. In wild-type worms, the YP170::EGFP endocytosed two nearly full-grown oocytes of one gonad arm (A, arrows). In obr quadruple mutants (obr-1;2;3;4), YP170::EGFP is efficiently endocytosed and stored in oocytes in a similar manner to that in wild-type worms (C, arrows). Scale bars are 20 µm. (0.49 MB TIF) [file pgen.1001055.s008.tif]

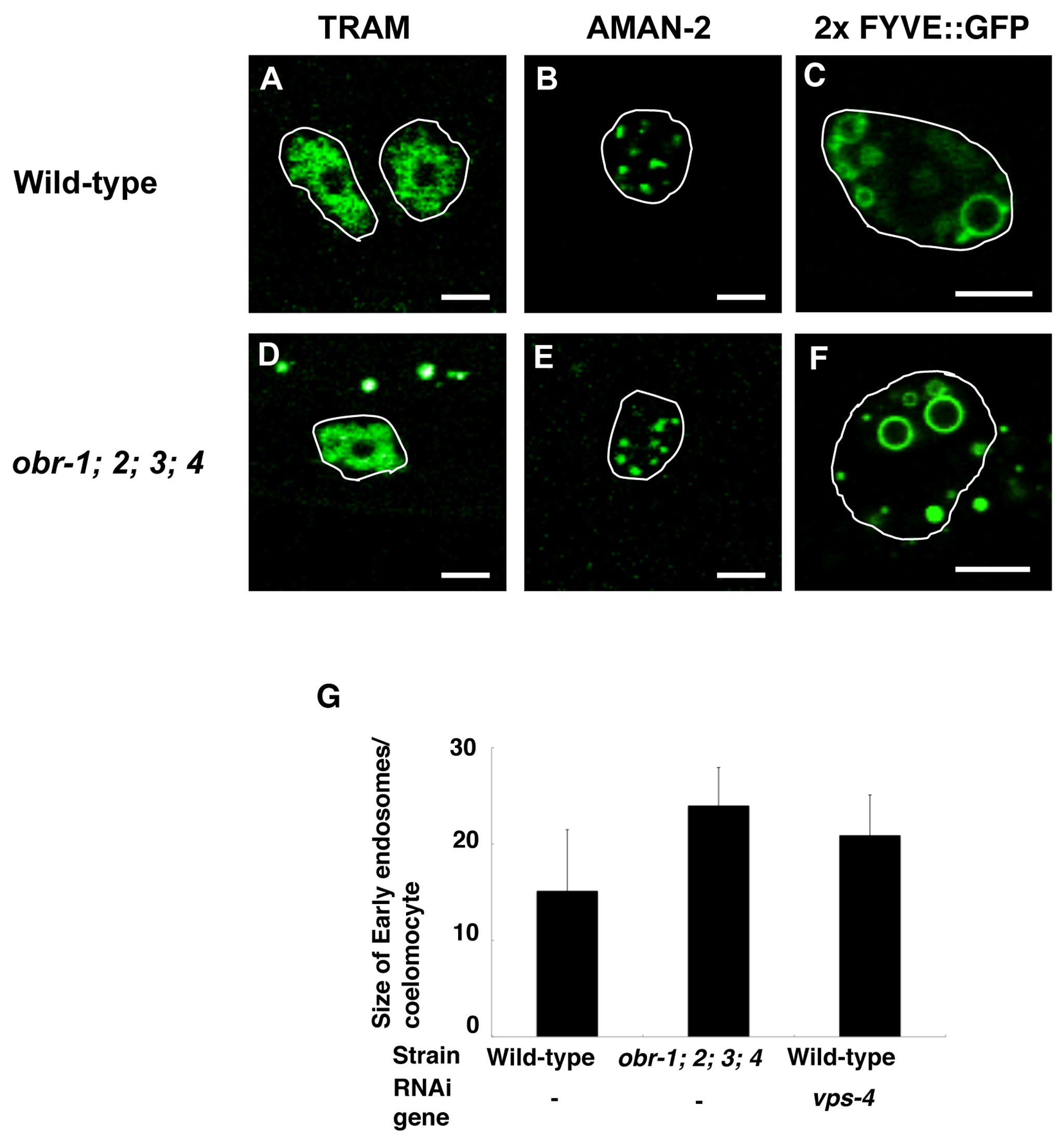

Supplement: Figure S9 — Morphology of ER, Golgi, and early endosomes is not affected in obr quadruple mutants. Confocal micrographs of wild-type and obr quadruple mutant coelomocytes (obr-1;2;3;4) expressing a GFP fusion organelle marker. TRAM; rER marker, AMAN-2 (mannosidase II); Golgi marker, 2xFYVE; early endosomal marker. The outline of the coelomocyte is indicated by a white line. All scale bars are 5 µm. (G) Quantification of the size of early endosomes in wild-type, obr quadruple mutants and vps-4 (RNAi) coelomocytes. The vertical axis indicates the ratio of early endosomal area per coelomocyte area. (0.72 MB TIF) [file pgen.1001055.s009.tif]

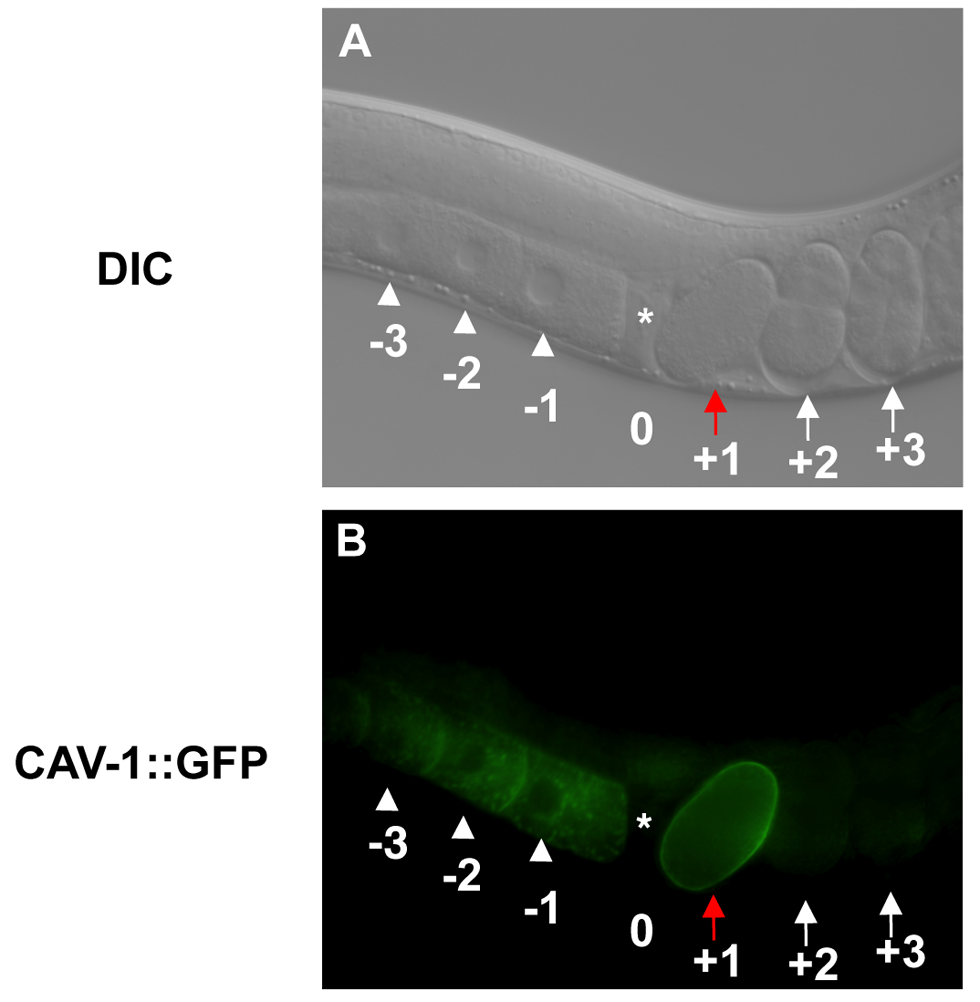

Supplement: Figure S10 — CAV-1::GFP is degraded after fertilization. (A and B) Normarski (A) and fluorescence (B) micrographs of wild-type hermaphrodites expressing CAV-1::GFP. In the proximal gonad, oocytes undergo maturation (A, arrowheads) and are ovulated into the sperm-containing spermatheca (A, asterisk) where they are fertilized. Fertilized eggs then move into the uterus (A, arrows). In control oocytes prior to fertilization, CAV-1::GFP is concentrated in intracellular vesicles and large ring-like cytoplasmic structures and localized weakly to the plasma membrane (A and B, arrowheads). Immediately after oocytes pass through the spermatheca and are fertilized, the amount of CAV-1::GFP on the cell surface rapidly increases, followed by its internalization and degradation. Newly fertilized embryos exhibited bright CAV-1::GFP fluorescence, initially at the cell surface (A and B, red arrows) and subsequently on internal membranes, but embryos beyond the 2-cell stage, approximately 90 minutes post fertilization, lacked visible fluorescence (A and B, white arrows). (0.26 MB TIF) [file pgen.1001055.s010.tif]

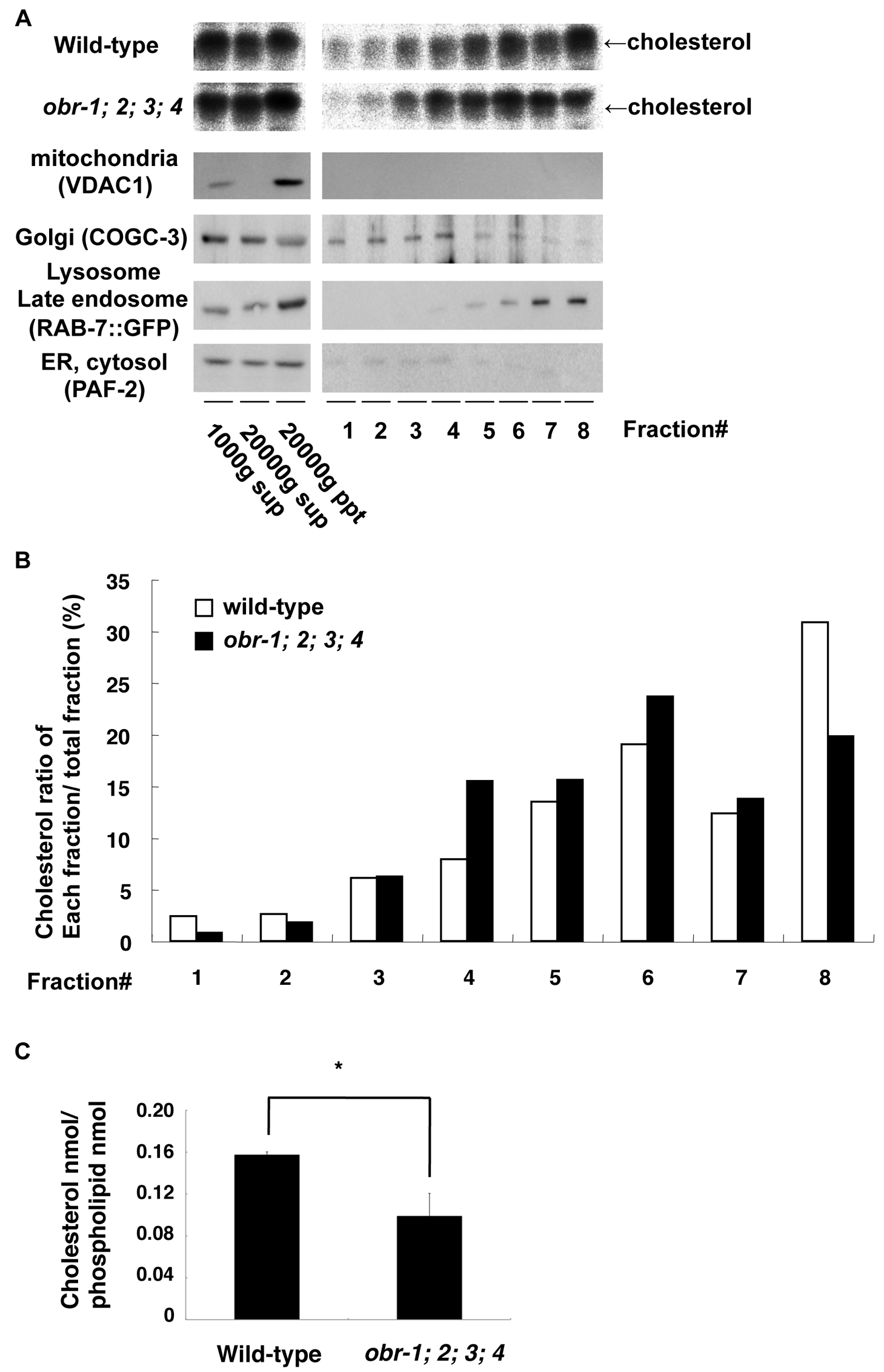

Supplement: Figure S11 — Late endosomal/lysosomal cholesterol is reduced in obr quadruple mutants. (A) Wild-type and obr quadruple mutants (obr-1;2;3;4) were disrupted with a Dounce homogenizer and the membrane fractions (20,000g ppt) were subjected to continuous OptiPrep density-gradient centrifugation (for details, see Materials and Methods). Aliquots of 1,000g sup, 20,000g sup, 20,000g ppt, and gradient fractions 1–8 were analyzed by immunoblotting using antibodies against the indicated proteins [1],[2]. The late endosomal/lysosomal fractions of worms were found at fractions 7 and 8. Lipids of each fraction were extracted and analyzed by TLC. The band corresponding to cholesterol was measured. (B) The amount of cholesterol in each fraction was quantified by densitometry and expressed as the percentage of cholesterol content of 20,000 g ppt. Similar data showing reduced cholesterol content in late endosomal/lysosomal fractions were obtained from two independent experiments. (C) Total cholesterol content in wild-type and obr quadruple mutants. Cholesterol amounts are expressed as nanomoles of cholesterol per nanomole of phospholipids. (0.57 MB TIF) [file pgen.1001055.s011.tif]

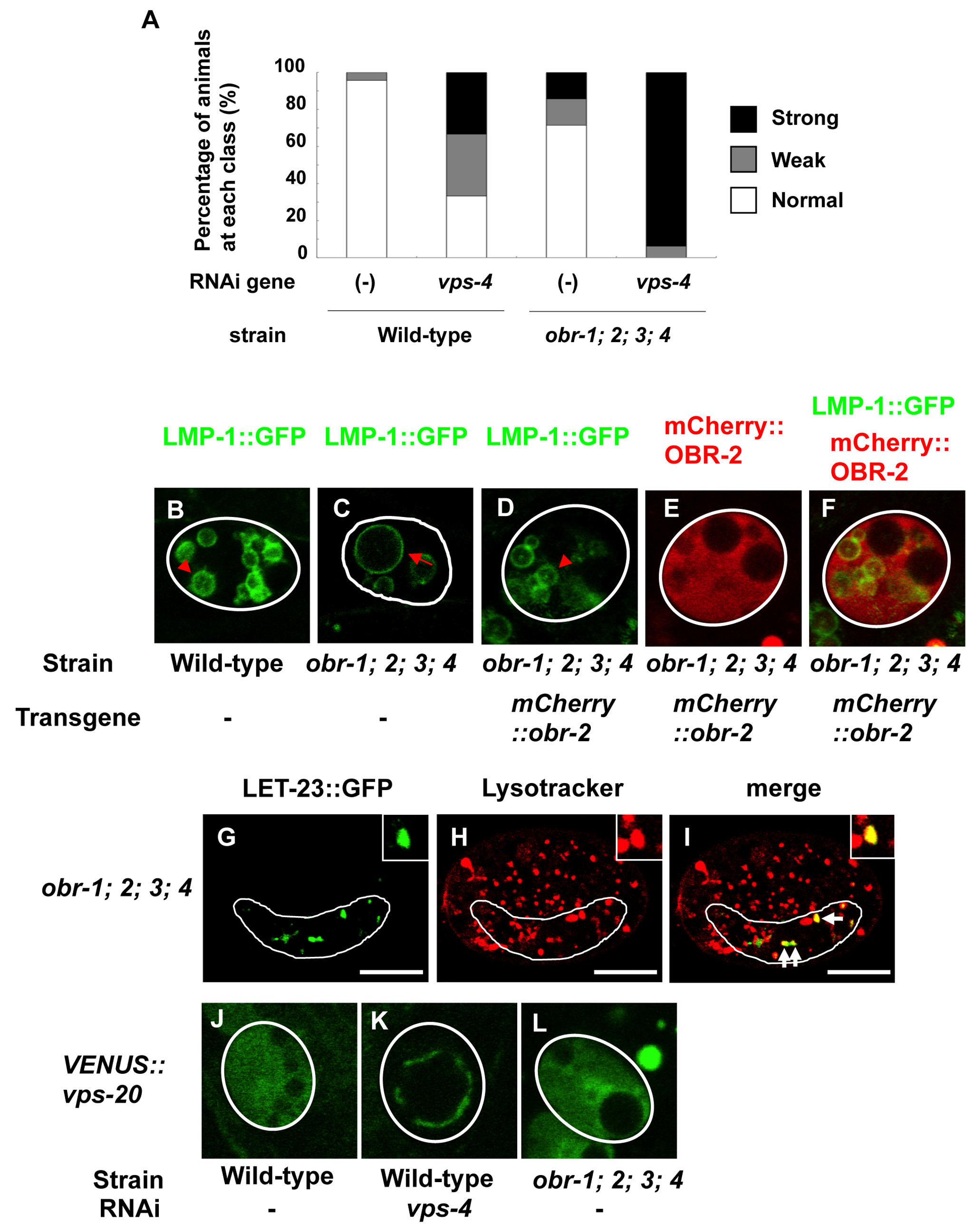

Supplement: Figure S12 — LET-23::GFP localized in enlarged endosomes/lysosomes.(A) Synergism between obr genes and vps-4. Diameter of LysoSensor-positive vesicles is classified into three categories: Normal (normal: <1.5 µm), Weak (weakly enlarged vesicle: 1.5–2 µm) and Strong (strongly enlarged: >2 µm). Graph shows the percentage of worms containing each category of LysoSensor-positive vesicles. (B–F) Expression of mCherry::OBR-2 fully rescues the enlarged late-endosomes/lysosomes in coelomocytes of obr quadruple mutants. (B–F) Confocal micrographs of coelomocytes expressing LMP-1::GFP. Wild-type (B), obr quadruple mutants (C), and obr quadruple mutants expressing mCherry::OBR-2 under the control of coelomocyte-specific unc-122 promoter (D). An arrow indicates abnormally enlarged lysosomes, and arrowheads indicate normal lysosomes. (E, F) Subcellular localization of OBR-2. mCherry::OBR-2 mainly localized in the cytosol. Note that expression of mCherry::OBR-2 fully rescues the enlarged lysosomes in obr quadruple mutant coelomocyes. The outlines of the coelomocytes are indicated by a white line. (G–I) obr quadruple mutants accumulate LET-23::GFP, a C. elegans EGF receptor. Confocal micrographs of embryos in obr quadruple mutants carrying the LET-23::GFP transgene [dpy-7p::let-23cDNA::GFP]. The transgenic worms were grown on plates containing Lysotracker red. The area enclosed by the white line indicates the epithelial cells which express LET-23::GFP. (H) Lysotracker red-positive vesicles out of the enclosed line are lysosomes in the cells which do not express LET-23::GFP (mainly intestinal cells and muscle cells). Note that most of the enlarged LET-23::GFP-positive vesicles are stained with Lysotracker red (I, arrows). (J–L) Confocal fluorescence images of wild-type (J), vps-4 (RNAi) (K), and obr quadruple mutants expressing VENUS::VPS-20 (L). The outline of the coelomocyte is indicated by a white line. In wild-type worms, VENUS::VPS-20 is localized in the cytosol. In contrast, VENUS::VPS-20 [file pgen.1001055.s012.tif]

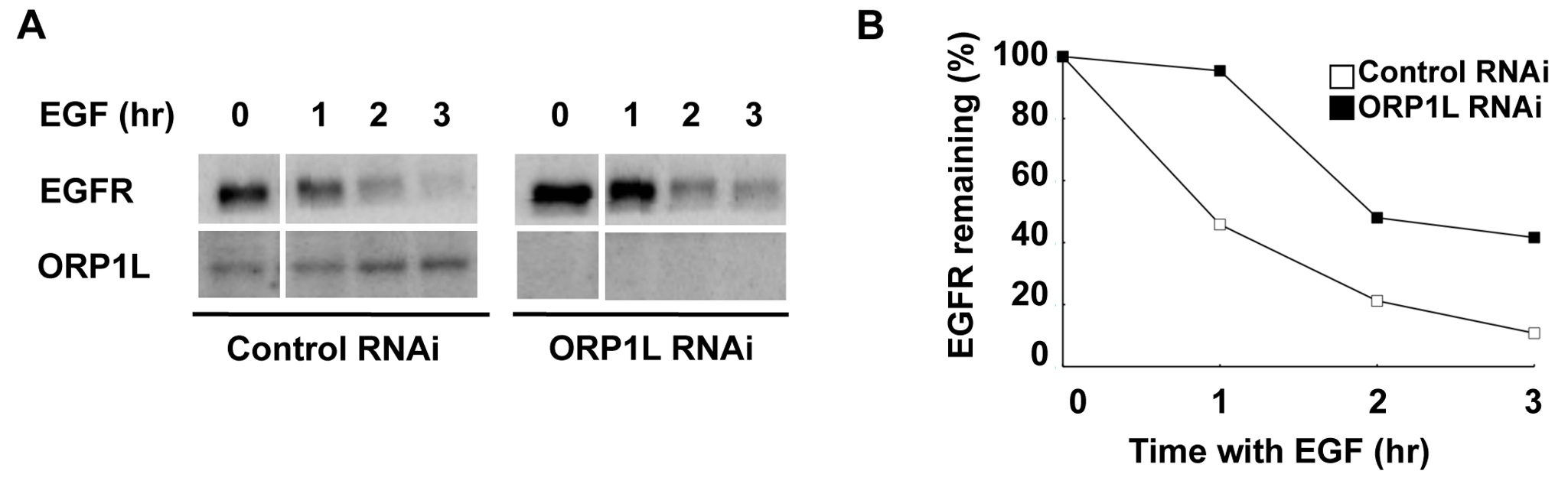

Supplement: Figure S13 — Depletion of ORP1L delays EGF receptor degradation. (A) HeLa cells (control RNAi or ORP1L RNAi) were treated with EGF (100 ng/ml) at 37°C for the periods indicated and the lysates were subjected to Western blot analysis with an anti-EGF receptor antibody. (B) The remaining EGF receptor bands at each time point were quantitated and indicated as a percentage relative to that at time 0 hr. (0.22 MB TIF) [file pgen.1001055.s013.tif]
