# Supplementary material for: Multivesicular Body Formation Requires OSBP–Related Proteins and Cholesterol
Source: PLoS Genet. 2010 Aug 5;6(8):e1001055. doi: 10.1371/journal.pgen.1001055 (PMC2916882; doi:10.1371/journal.pgen.1001055)
Supplement: Table S1 — A list of genes tested for synthetic lethality with obr quadruple mutations. (0.13 MB DOC) [file pgen.1001055.s014.doc]

**Table S1.** A List of genes tested for synthetic lethality with *obr* quadruple mutations.

| **Systematic name** | **Standard name** | **Structural description** | **Functional group** |
| --- | --- | --- | --- |
| **C34G6.7** |  | **ESCRT 0** | **MVB formation** |
| **C07G1.5** | ***hgrs-1*** | ESCRT 0 | **MVB formation** |
| **C09G12.9** |  | ESCRT I | **MVB formation** |
| **Y87G2A.10** |  | **ESCRT I** | **MVB formation** |
| **C27F2.5** |  | **ESCRT II** | **MVB formation** |
| **W02A11.2** |  | **ESCRT II** | **MVB formation** |
| **F17C11.8** |  | **ESCRT II** | **MVB formation** |
| **Y46G5A.12** |  | **ESCRT III** | **MVB formation** |
| **Y65B4A.3** |  | **ESCRT III** | **MVB formation** |
| **T27F7.1** |  | **ESCRT III** | **MVB formation** |
| **C37C3.3** |  | **ESCRT III** | **MVB formation** |
| **C56C10.3 *^a^** |  | **ESCRT III** | **MVB formation** |
| **Y34D9A.10** |  | **AAA ATPase** | **MVB formation** |
| **F23C8.6** |  | **DID2** | **MVB formation** |
| **F41E6.9** |  | **vps60** | **MVB formation** |
| **R10E12.1** |  | **Bro1** | **MVB formation** |
| **C39F7.4 *** |  | **Rab1** | **Small G protein** |
| **F53F10.4** |  | **Rab2** | **Small G protein** |
| **F11A5.3** |  | **Rab2** | **Small G protein** |
| **F11A5.4** |  | **Rab2** | **Small G protein** |
| **C18A3.6** |  | **Rab3** | **Small G protein** |
| **F26H9.6 *** |  | **Rab5** | **Small G protein** |
| **F59B2.7** |  | **Rab6** | **Small G protein** |
| **T25G12.4** |  | **Rab6** | **Small G protein** |
| **W03C9.3** |  | **Rab7** | **Small G protein** |
| **D1037.4** |  | **Rab8** | **Small G protein** |
| **T23H2.5** |  | **Rab10** | **Small G protein** |
| **F53G12.1** |  | **Rab11** | **Small G protein** |
| **W04G5.2** |  | **Rab11** | **Small G protein** |
| **K09A9.2** |  | **Rab14** | **Small G protein** |
| **T01B7.3** |  | **Rab21** | **Small G protein** |
| **ZK669.5** |  | **Rab23** | **Small G protein** |
| **Y11D7A.4** |  | **Rab28** | **Small G protein** |
| **W01H2.3** |  | **Rab37** | **Small G protein** |
| **K02E10.1** |  | **Rab37** | **Small G protein** |
| **D2013.1** |  | **Rab39** | **Small G protein** |
| **ZK632.8** |  | **Arl5** | **Small G protein** |
| **C38D4.8** |  | **Arl6** | **Small G protein** |
| **ZK180.4 *** |  | **Sar1b** | **Small G protein** |
| **F19H8.3** |  | **Arl3** | **Small G protein** |
| **Y116A8C.12** |  | **Arf6** | **Small G protein** |
| **F20D1.5** |  | **Arl7** | **Small G protein** |
| **B0336.2** |  | **Arf1** | **Small G protein** |
| **F54C9.10 *** |  | **Arl1** | **Small G protein** |
| **F54E4.1** |  | **RAVE** | **Small G protein** |
| **F57H12.1 *** |  | **Arf5** | **Small G protein** |
| **F22B5.1** |  | **Arl2** | **Small G protein** |
| **C06G3.10** |  | **COG2** | **COG complex** |
| **C43E11.11** |  | **COG5** | **COG complex** |
| **K07C11.9** |  | **COG6** | **COG complex** |
| **R02D3.2** |  | **COG8** | **COG complex** |
| **B0361.10** |  | **Ykt6** | **SNARE** |
| **F55A11.2 *** |  | **Syn5** | **SNARE** |
| **M03E7.5** |  | **GS27/Bos1/membrin** | **SNARE** |
| **B0272.2** |  | **GS27/Bos1/membrin** | **SNARE** |
| **T10H9.3** |  | **Syn18** | **SNARE** |
| **F48F7.2** |  | **Syn1c** | **SNARE** |
| **T01B11.3** |  | **Syn1d** | **SNARE** |
| **C15C7.1** |  | **Syn6** | **SNARE** |
| **VF39H2l.1** |  | **Syn7** | **SNARE** |
| **ZC155.7** |  | **Syn16** | **SNARE** |
| **Y22F5A.3** |  | **Snap25** | **SNARE** |
| **K02D10.5 *** |  | **Snap29** | **SNARE** |
| **T10H9.4** |  | **VAMP1** | **SNARE** |
| **F23H12.1** |  | **VAMP2** | **SNARE** |
| **C30A5.5** |  | **VAMP3** | **SNARE** |
| **F55A4.1** |  | **Sec22b** | **SNARE** |
| **F27D9.1** |  | **nsec1** | **SEC1 family** |
| **T07A9.10** |  | **Munc18b** | **SEC1 family** |
| **F43D9.3 *** |  | **Sly1** | **SEC1 family** |
| **B0303.9** |  | **vps33a** | **SEC1 family** |
| **C56C10.1** |  | **vps33b** | **SEC1 family** |
| **C44C1.4** |  | **vps45** | **SEC1 family** |
| **F29G9.3 *** |  | **AP1** | **AP complex** |
| **F02E8.3 *** |  | **AP2** | **AP complex** |
| **F55A12.7** |  | **AP1** | **AP complex** |
| **K11D2.3** |  | **AP1** | **AP complex** |
| **R160.1** |  | **AP2** | **AP complex** |
| **F53H8.1** |  | **AP3** | **AP complex** |
| **R11A5.1** |  | **AP3** | **AP complex** |
| **T20B5.1** |  | **AP2** | **AP complex** |
| **W09G10.4a** |  | **AP3** | **AP complex** |
| **T20G5.1 *** |  | **Clathrin heavy chain1** | **Clathrin** |
| **T05B11.3** |  | **Clathrin Light Chain A** | **Clathrin** |
| **Y25C1A.5 *** |  | **Beta subunit of COP I complex** | **COP I component** |
| **F38E11.5 *** |  | **Beta’ subunit of COP I complex** | **COP I component** |
| **T14G10.5 *** |  | **Gamma subunit of COP I complex** | **COP I component** |
| **C13B9.3 *** |  | **Delta subunit of COP I complex** | **COP I component** |
| **F45G2.4 *** |  | **Epsilon subunit of COP I complex** | **COP I component** |
| **F59E10.3 *** |  | **Zeta subunit of COP I complex** | **COP I component** |
| **Y113G7A.3 *** |  | **Sec 23A** | **COP II component** |
| **ZC518.2** |  | **Sec 24A** | **COP II component** |
| **F12F6.6 *** |  | **Sec 24A** | **COP II component** |
| **T01G1.3** |  | **Sec 31A** | **COP II component** |
| **C05D9.1** |  | **Vps 5** | **Retromer complex** |
| **T20D3.7** |  | **Vps26A** | **Retromer complex** |
| **ZK1128.8** |  | **Vps29** | **Retromer complex** |
| **F59G1.3** |  | **Vps35** | **Retromer complex** |
| **C36B1.4** |  | **Proteasome subunit** | **Proteasome** |
| **F25H2.9 *** |  | **Proteasome subunit** | **Proteasome** |
| **D1054.2** |  | **Proteasome subunit** | **Proteasome** |
| **CD4.6 *** |  | **Proteasome subunit** | **Proteasome** |

^a^Asterisks indicate genes whose knockdown resulted in sterility or high embryonic lethality in wild-type animals.
