# Supplementary material for: Multivesicular Body Formation Requires OSBP–Related Proteins and Cholesterol
Source: PLoS Genet. 2010 Aug 5;6(8):e1001055. doi: 10.1371/journal.pgen.1001055 (PMC2916882; doi:10.1371/journal.pgen.1001055)
Supplement: Table S2 — Genetic enhancers of obr quadruple mutants. (0.08 MB DOC) [file pgen.1001055.s015.doc]

**Table S2. Genetic enhancers of *obr* quadruple mutants.**

|  | **Linkage group** | **Systematic name** | **Standard name** | **Structural description** | **Mammalian homologue** | **Functional group** |
| --- | --- | --- | --- | --- | --- | --- |
| **Group A^a^** | *I* | **Y34D9A.10** | ***vps-4*** | **AAA ATPase** | **VPS4 (3.6e-161^c^)** | **MVB formation** |
|  | ***I*** | **Y87G2A.10** | ***vps-28*** | **ESCRT I** | **VPS28 (1.6e-46)** | **MVB formation** |
|  | ***I*** | **Y65B4A.3** | ***vps-2*** | **ESCRT III** | **CHMP6(3.9e-45)** | **MVB formation** |
|  | ***I*** | **B0025.1** | ***vps-34*** | **PI 3 kinase** | **PI3 kinase type3 (7.8e-150)** | **PI(3)P synthase** |
|  | ***III*** | **F55A12.7** | ***apm-1*** | **mu1-II subunit of adaptor protein complex 1** | **AP-1 complex subunit mu-1 (1.1e-168)** | **AP complex** |
|  | ***III*** | **C38D4.5** | ***tag-325*** | **Predicted Rho GTPase-activating protein** | **66 kDa protein (7e-59)** | **RHO GAP** |
|  | ***III*** | **B0336.2** | ***arf-1.2*** | **Arf1 (GTP-binding ADP-ribosylation factor)** | **ADP-ribosylation factor 1 (2.8e-90)** | **Small G protein** |
|  | ***III*** | **ZK328.1** | ***cyk-3*** | **Ubiquitin C-terminal hydrolase** | **Ubiquitin C-terminal hydrolase 32 (1.5e-97)** | **Ub related** |
|  | ***I*** | **C10H11.9** | ***let-502*** | **Rho-associated protein kinase** | **Rho-associated kinase 1 (6.81e-158)** | **Signaling** |
|  | ***III*** | **B0280.11** |  | **Receptor-type tyrosine-protein phosphatase** | **Receptor-type PTP phosphatase (4.2e-11)** | **Signaling** |
|  | ***III*** | **F54E7.3** | ***par-3*** | **PDZ domain-containing protein** | **Par-3 homologue (7.2e-38)** | **Signaling** |
|  | ***I*** | **F28B3.7** | ***him-1*** | **SMC1 (structural maintenance of chromosomes) protein** | **SMC protein 1A (1.9e-267)** | **Nuclear protein** |
|  | ***I*** | **C32F10.5** | ***hmg-3*** | **Nucleosome-binding factor SPN, POB3** | **FACT complex subunit (3e-134)** | **Nuclear protein** |
|  | ***III*** | **E03A3.3** | ***his-69*** | **Histones H3 and H4** | **Histone H3.2 (4.4e-53)** | **Nuclear protein** |
|  | ***III*** | **R07E5.10** | ***pdcd-2*** | **Uncharacterized MYND Zn-finger protein** | **Programmed cell death protein 2 (8.2e-36)** | **Nuclear protein** |
|  | ***III*** | **T20B12.1** |  | **Tetratricopeptide repeat protein** | **TRP repeat protein 27 (1.3e-90)** | **Unknown** |
|  | ***III*** | **R13F6.1** | ***kbp-1*** | **KNL (kinetochore null) Binding Protein** | **Unknown** | **Unknown** |
|  | ***III*** | **C45G9.5** |  |  | **Unknown** | **Unknown** |
|  | ***III*** | **R144.3** |  |  | **Unknown** | **Unknown** |
|  | ***III*** | **F08F8.2** |  | **HMG-CoA reductase** | **HMG-CoA reductase (1.7e-106)** | **Metabolism** |
|  | ***III*** | **C05D11.12** | ***let-721*** | **ubiquinone oxidoreductase** | **flavoprotein-ubiquinone oxidoreductase (6.4e-203)** | **Metabolism** |
|  | ***III*** | **F37C12.9** | ***rps-14*** | **40S ribosomal protein** | **40S ribosomal protein (7.3e-67)** | **Ribosome** |
| **Group B^b^** | *II* | **Y46G5A.12** | ***vps-20*** | **ESCRT III** | **CHMP2a (1.2e-75)** | **MVB formation** |
|  | ***IV*** | **C07G1.5** | ***hgrs-1*** | **ESCRT 0** | **HRS (1.2e-107)** | **MVB formation** |
|  | ***II*** | **ZK930.1** |  | **Protein kinase containing WD40 repeats** | **PI 3-kinase regulatory subunit 4 (1.5e-111)** | **PI(3)P synthase** |
|  | ***II*** | **F52C6.12** |  | **Ubiquitin-conjugating enzyme E2** | **Ubiquitin-conjugating enzyme (3e-22)** | **Ub related** |
|  | ***X*** | **R160.1** | ***dpy-23*** | **mu2 subunit of adaptor protein complex 2** | **AP-2 complex subunit mu-2 (2.4e-196)** | **AP complex** |
|  | ***X*** | **T20B5.1** | ***apa-2*** | **alpha subunit of adaptor protein complex 2** | **AP-2 complex subunit alpha-1 (0)** | **AP complex** |

^a^Group A, genes on chromosomes I and III.

^b^Group B, genes involved in intracellular vesicular transport.

^c^E-value.
