# Supplementary material for: Multivesicular Body Formation Requires OSBP–Related Proteins and Cholesterol
Source: PLoS Genet. 2010 Aug 5;6(8):e1001055. doi: 10.1371/journal.pgen.1001055 (PMC2916882; doi:10.1371/journal.pgen.1001055)
Supplement: Text S1 — Supporting materials. (0.06 MB DOC) [file pgen.1001055.s016.doc]

**SUPPORTING INFORMATION**

**SUPPORTING MATERIALS**

**Preparation of transgenic worms**

DNA injection into the *C.* *elegans* germ line was carried out as described [3]. The array *xhEx2503[obr-2genome::GFP]*, *xhEx2511[unc122p::mCherry::obr-1cDNA]*, *xhEx2512[unc122p::mCherry::obr-2cDNA]*, *xhEx2513[unc122p::mCherry::obr-3cDNA]*, *xhEx2514[unc122p::mCherry::obr-4cDNA]*, *xhIs2501[dpy-7p::let-23cDNA::GFP]* , *xhEx2515[unc-122p::venus::vps-20cDNA]* contained the plasmids pHK3 (*obr-2genome::GFP*), pHK7 (*dpy-7p::let-23cDNA::GFP*), pHK9 (*unc122p::mCherry::obr-1cDNA*), pHK10 (*unc122p::mCherry::obr-2cDNA*), pHK11 (*unc122p::mCherry::obr-3cDNA*), pHK12 (*unc122p::mCherry::obr-4cDNA*), and pHK26 (*unc122p::venus::vps-20cDNA*), respectively. pHK3, pHK7, pHK9, pHK10, pHK11, pHK12, and pHK26 were prepared as follows.

pHK3; A 7,249 bp genomic fragment containing a 5,000 bp upstream of the initiation codon and part of the coding sequence truncated at the 3’ end was amplified by PCR from wild-type worms with primers *obr-2 genome*-F, 5’-TAA AAT CTG CAG TGA AAT CCA AAA TTT GTC GG-3’ and *obr-2* *genome*-R, 5’-TTC TGA CCC GGG AAT CGA CTG AAG AAA TTG ATG A-3’. The fragment was cloned in frame with the GFP gene into the pPD95.67 vector at the *Pst*I and *Sma*I sites.

pHK7; Full-length *let-23* cDNA was amplified by PCR from a *C. elegans* cDNA library with primers *let-23*-F1, 5’-TTC TGC CCG GGA TGC GAT ACC CTC CCT CTA TC-3’ and *let-23*-R1, 5’-GTT CGG CGG CCG CAA GAC AAG TTT CCT TTT GTG-3’. The fragment was cloned into the pTK030 vector at the *Sma*I and *Not*I sites.

pHK9, pHK10, pHK11 and pHK12; Full-length *obr* cDNA was amplified by polymerase chain reaction (PCR) from a *C. elegans* cDNA library, and integrated into the *Bgl*II site of *pFX_unc-122p::mCherry* vector using an In-Fusion Dry-down PCR Cloning Kit (Clontech). The following primers were used for cloning of *obr-1*, *obr-2*, *obr-3* and *obr-4* cDNA: *obr-1*-F, 5’-TTG TAT AAG AGA TCT ATG CCG TCG CTT TCA GAA TT-3’; *obr-1*-R, 5’- TAC TTC AAA AAT AGA CTA AAA AAT ATC GGG GCA CT-3’; *obr-2*-F, 5’-TTG TAT AAG AGA TCT ATG GTG AAG CTT TTC AGA GA-3’; *obr-2*-R, 5’-TAC TTC AAA AAT AGA TCA ATC GAC TGA AGA AAT TG-3’; *obr-3*-F, 5’-TTG TAT AAG AGA TCT ATG GAG GAA TTT GAC GAA AA-3’; *obr-3*-R, 5’-TAC TTC AAA AAT AGA TCA CTT GCG AAG CAT GAA GA-3’; *obr-4*-F, 5’-TTG TAT AAG AGA TCT ATG GAA GGT CCG CTC TCA AA-3’; *obr-4*-R, 5’- TAC TTC AAA AAT AGA TCA AAC TGC TTT TCT GAC AC-3’.

pHK26; Full-length *vps-20* cDNA was amplified by polymerase chain reaction (PCR) from a *C. elegans* cDNA library, and integrated into the *Bgl*II site of *pFX_unc-122p::venus* vector using an In-Fusion Dry-down PCR Cloning Kit (Clontech). The following primers were used for cloning of *vps-20* cDNA: *vps-20*-F, 5’- GCT GTA CAA GAG ATC TAT GGG TGG AAT TTT CTC GAA-3’; *vps-20*-R, 5’- TCA AAA ATA GAG ATC TTT AGG CTT CCA GAG CAA TCT-3’.

**Total Cholesterol content analysis**

Lipids were extracted from mixed stage worms by the method of Bligh and Dyer. Total cholesterol content was measured by the Amplex Red Cholesterol Assay Kit (Molecular Probes, A-12216).

**EGF receptor degradation assay**

Cells were transfected for 48 hr with 20nM ORP1L-specific (sense strand GGACGAAAGGAGUUGGUAAdTdG) or control siRNA (Nippon EGT, Japan) using Lipofectamine 2000 and then serum-starved for 1 hr. Thereafter, cells were treated with 100 ng/ml EGF. At the indicated times, cells were harvested and the whole-cell lysates were subjected to Western blot analysis with an anti-EGF receptor antibody.

**SUPPORTING REFERENCES**

1. Inoue T, Sugimoto A, Suzuki Y, Yamamoto M, Tsujimoto M, et al. (2004) Type II platelet-activating factor-acetylhydrolase is essential for epithelial morphogenesis in Caenorhabditis elegans. Proc Natl Acad Sci U S A 101: 13233-13238.

2. Kubota Y, Sano M, Goda S, Suzuki N, Nishiwaki K (2006) The conserved oligomeric Golgi complex acts in organ morphogenesis via glycosylation of an ADAM protease in C. elegans. Development 133: 263-273.

3. Mello C, Kramer J, Stinchcomb D, Ambros V (1991) Efficient gene transfer in C.elegans: extrachromosomal maintenance and integration of transforming sequences. EMBO J 10: 3959-3970.

**Figure S1. ORP Family in *H. sapiens*, *C. elegans*, and *S. cerevisiae*.**

The *H. sapiens*, *C. elegans*, and *S. cerevisiae* ORP families. Domain structures of the major variants are shown. The human proteins can be subdivided into six subfamilies (indicated with Roman numerals) based on gene structure and amino acid homology. In *C. elegans*, 4 ORP members are conserved (OBR-1, OBR-2, OBR-3, OBR-4) and classified into the subfamilies I, II, IV, and V, respectively. Yeast ORP members (OSH1 to Osh7) share comparatively low sequence homologies with mammalian ORP proteins. Blue box, PH domain; red box, sterol binding domain; yellow box, EQVSHHPP motif which is fully conserved in all members of the family; green box, hydrophobic region; tangerine box, ankyrin-repeat; pink box, Golgi dynamics domain.

**Figure S2. Gene structures of *obr-1*, *obr-2*, *obr-3* and *obr-4*.**

Genomic structures of *obr-1*, *obr-2*, *obr-3* and *obr-4*. Boxes represent exons. The start (ATG) and stop (TAG or TGA) codons are indicated above the ﬁrst and last exons of each gene. The EQVSHHPP motif, which is completely conserved in all ORP family proteins, is indicated in yellow. Red, blue, and green indicate the regions encoding the sterol-binding domain, PH domain, and hydrophobic putative transmembrane domain. The extent of the deletion in *obr-1(xh16)*, *obr-2(xh17)*, *obr-3(tm1087)*, and *obr-4(tm1567)* is indicated by a horizontal line. *obr-1(xh16)* and *obr-3(tm1087)* contain 1716-bp and 613-bp deletions, respectively, in their sterol-binding domains. *obr-1(xh16)* allele lacks an ORP signature “EQVSHHPP” motif. *obr-3(tm1087)* harbors an in-frame deletion located 125 amino acids downstream of its “EQVSHHPP” motif and lacks 22 amino acids in the sterol-binding domain. *obr-2(xh17)* is a 1724 bp deletion and removes the *N*-terminal half of the protein including its ATG initiation codon. *obr-4(tm1567)* possesses a 540-bp deletion which causes a premature stop codon, resulting in a truncated protein lacking the sterol-binding domain.

**Figure S3. Structure of subfamily I ORPs.**

Multiple sequence alignment of the conserved sterol-binding domain of the *C. elegans* OBR-1 and homologous sequences in *D. melanogaster* (dobr-1), human (hOSBP, hORP4), and mouse (mOSBP, mORP4). Sequences were aligned with Clustal W. Residues identical, or related, in three or more of the sequences are indicated by black or gray boxes, respectively. The number on the right indicates amino acid positions. The EQVSHHPP motif is underlined in yellow. Accession numbers for the sequences used were as follows: *C. elegans* OBR-1: NP_499448; *D. melanogaster* OBR-1: NP_477271; human OSBP: NP_002547; mouse OSBP: NP_001028346; human ORP4: NP_110385; mouse ORP4: NP_690031.

**Figure S4. Structure of subfamily II ORPs.**

Multiple sequence alignment of the conserved sterol-binding domain of the *C. elegans* OBR-2 and homologous sequences in *D. melanogaster* (dobr-2), human (hORP1L, hORP2), and mouse (mORP1L, mORP2). The EQVSHHPP motif is underlined in yellow. Accession numbers for the sequences used were as follows: *C. elegans* OBR-2: NP_506695; *D. melanogaster* OBR-2: NP_611865; human ORP1L: NP_542164; mouse ORP1L: NP_997413; human ORP2: NP_653081; mouse ORP2: NP_653083.

**Figure S5. Structure of subfamily IV ORPs.**

Multiple sequence alignment of the conserved sterol-binding domain of the *C. elegans* OBR-3 and homologous sequences in *D. melanogaster* (dobr-3), human (hORP5, hORP8), and mouse (mORP5, mORP8). The EQVSHHPP motif is underlined in yellow. Accession numbers for the sequences used were as follows: *C. elegans* OBR-3: NP_741923; *D. melanogaster* OBR-3: NP_650878; human ORP5: NP_065947; mouse ORP5: NP_077251; human ORP8: NP_065892; mouse ORP8: NP_780698.

**Figure S6. Structure of subfamily V ORPs.**

Multiple sequence alignment of the conserved sterol-binding domain of the *C. elegans* OBR-4 and homologous sequences in *D. melanogaster* (dobr-4), human (hORP9), and mouse (mORP9). The EQVSHHPP motif is underlined in yellow. Accession numbers for the sequences used were as follows: *C. elegans* OBR-4: NP_491691; *D. melanogaster* OBR-4: NP_610534; human ORP9: NP_078862; mouse ORP9: NP_598646.

**Figure S7. *obr* quadruple mutants exhibit abnormal hypodrmis and cuticle.**

Transmission electron micrographs of wild-type (A and C) and *obr* quadruple mutants (B and D). (A-D) Transverse sections through the cuticle. In wild-type worms, the three ridges of the alae are observed (A, arrowheads), and the cuticle is approximately 0.5 μm in thickness with a flat surface (C, arrowheads). On the other hand, in *obr* quadruple mutants (*obr-1;2;3;4*), the morphology of alae is severely affected (B, arrows), the cuticle's outer surface is wavy instead of flat (D, arrows). Note that *obr* quadruple mutants have enlarged vacuoles which are not observed in wild-type worms (B, asterisks). Scale bar represents 2 μm.

**Figure S8. *obr* quadruple mutants exhibit no abberation with receptor-mediated endocytosis.**

(A-D) Fluorescence images and the corresponding Nomarski micrographs of adult hermaphrodites of wild-type and *obr* quadruple mutants carrying the YP170::EGFP transgene. The YP170::EGFP fusion protein is transported like endogenous yolk, from intestine to oocyte via receptor-mediated endocytosis. In wild-type worms, the YP170::EGFP endocytosed two nearly full-grown oocytes of one gonad arm (A, arrows). In *obr* quadruple mutants (*obr-1;2;3;4*), YP170::EGFP is efficiently endocytosed and stored in oocytes in a similar manner to that in wild-type worms (C, arrows). Scale bars are 20 μm.

**Figure S9. Morphology of ER, Golgi, and early endosomes is not affected in *obr* quadruple mutants.**

Confocal micrographs of wild-type and *obr* quadruple mutant coelomocytes (*obr-1;2;3;4*) expressing a GFP fusion organelle marker. TRAM; rER marker, AMAN-2 (mannosidase II); Golgi marker, 2xFYVE; early endosomal marker. The outline of the coelomocyte is indicated by a white line. All scale bars are 5 μm. (G) Quantification of the size of early endosomes in wild-type, *obr* quadruple mutants and *vps-4 (RNAi)* coelomocytes. The vertical axis indicates the ratio of early endosomal area per coelomocyte area.

**Figure S10. CAV-1::GFP is degraded after fertilization.**

(A and B) Normarski (A) and fluorescence (B) micrographs of wild-type hermaphrodites expressing CAV-1::GFP. In the proximal gonad, oocytes undergo maturation (A, arrowheads) and are ovulated into the sperm-containing spermatheca (A, asterisk) where they are fertilized. Fertilized eggs then move into the uterus (A, arrows). In control oocytes prior to fertilization, CAV-1::GFP is concentrated in intracellular vesicles and large ring-like cytoplasmic structures and localized weakly to the plasma membrane (A and B, arrowheads). Immediately after oocytes pass through the spermatheca and are fertilized, the amount of CAV-1::GFP on the cell surface rapidly increases, followed by its internalization and degradation. Newly fertilized embryos exhibited bright CAV-1::GFP fluorescence, initially at the cell surface (A and B, red arrows) and subsequently on internal membranes, but embryos beyond the 2-cell stage, approximately 90 minutes post fertilization, lacked visible fluorescence (A and B, white arrows).

**Figure S11. Late endosomal/lysosomal cholesterol is reduced in *obr* quadruple mutants.**

(A) Wild-type and *obr* quadruple mutants (*obr-1;2;3;4*) were disrupted with a Dounce homogenizer and the membrane fractions (20,000g ppt) were subjected to continuous OptiPrep density-gradient centrifugation (for details, see Materials and Methods). Aliquots of 1,000g sup, 20,000g sup, 20,000g ppt, and gradient fractions 1-8 were analyzed by immunoblotting using antibodies against the indicated proteins [1,2]. The late endosomal/lysosomal fractions of worms were found at fractions 7 and 8. Lipids of each fraction were extracted and analyzed by TLC. The band corresponding to cholesterol was measured. (B) The amount of cholesterol in each fraction was quantified by densitometry and expressed as the percentage of cholesterol content of 20,000 g ppt. Similar data showing reduced cholesterol content in late endosomal/lysosomal fractions were obtained from two independent experiments. (C) Total cholesterol content in wild-type and *obr* quadruple mutants. Cholesterol amounts are expressed as nanomoles of cholesterol per nanomole of phospholipids.

**Figure S12. LET-23::GFP localized in enlarged endosomes/lysosomes.**

(A) Synergism between *obr* genes and *vps-4*. Diameter of LysoSensor-positive vesicles is classified into three categories: Normal (normal: < 1.5 μm), Weak (weakly enlarged vesicle: 1.5-2 μm) and Strong (strongly enlarged: > 2 μm). Graph shows the percentage of worms containing each category of LysoSensor-positive vesicles.

(B-F) Expression of mCherry::OBR-2 fully rescues the enlarged late-endosomes/lysosomes in coelomocytes of *obr* quadruple mutants. (B-F) Confocal micrographs of coelomocytes expressing LMP-1::GFP. Wild-type (B), *obr* quadruple mutants (C), and *obr* quadruple mutants expressing mCherry::OBR-2 under the control of coelomocyte-specific *unc-122* promoter (D). An arrow indicates abnormally enlarged lysosomes, and arrowheads indicate normal lysosomes. (E, F) Subcellular localization of OBR-2. mCherry::OBR-2 mainly localized in the cytosol. Note that expression of mCherry::OBR-2 fully rescues the enlarged lysosomes in *obr* quadruple mutant coelomocyes. The outlines of the coelomocytes are indicated by a white line.

(G-I) *obr* quadruple mutants accumulate LET-23::GFP, a *C. elegans* EGF receptor. Confocal micrographs of embryos in *obr* quadruple mutants carrying the LET-23::GFP transgene *[dpy-7p::let-23cDNA::GFP]*. The transgenic worms were grown on plates containing Lysotracker red. The area enclosed by the white line indicates the epithelial cells which express LET-23::GFP. (H) Lysotracker red-positive vesicles out of the enclosed line are lysosomes in the cells which do not express LET-23::GFP (mainly intestinal cells and muscle cells). Note that most of the enlarged LET-23::GFP-positive vesicles are stained with Lysotracker red (I, arrows).

(J-L) Confocal fluorescence images of wild-type (J), *vps-4 (RNAi)* (K), and *obr* quadruple mutants expressing VENUS::VPS-20 (L). The outline of the coelomocyte is indicated by a white line. In wild-type worms, VENUS::VPS-20 is localized in the cytosol. In contrast, VENUS::VPS-20 is translocated from the cytosol to the membrane-like structure, possibly enlarged lysosomes. In *obr* quadruple mutants, VENUS::VPS-20 is localized in the cytosol in a similar manner to that in wild-type worms.

**Figure S13. Depletion of ORP1L delays EGF receptor degradation.**

(A) HeLa cells (control RNAi or ORP1L RNAi) were treated with EGF (100 ng/ml) at 37°C for the periods indicated and the lysates were subjected to Western blot analysis with an anti-EGF receptor antibody. (B) The remaining EGF receptor bands at each time point were quantitated and indicated as a percentage relative to that at time 0 hr.
